# Caveolae disassemble upon membrane lesioning and foster cell survival

**DOI:** 10.1016/j.isci.2024.108849

**Published:** 2024-01-09

**Authors:** Martin Štefl, Masanari Takamiya, Volker Middel, Miyase Tekpınar, Karin Nienhaus, Tanja Beil, Sepand Rastegar, Uwe Strähle, Gerd Ulrich Nienhaus

**Affiliations:** 1 Institute of Applied Physics (APH), Karlsruhe Institute of Technology (KIT), Wolfgang Gaede-Strasse 1, 76131 Karlsruhe, Germany; 2 Institute of Biological and Chemical Systems (IBCS), Karlsruhe Institute of Technology (KIT), PO Box 3640, 76021 Karlsruhe, Germany; 3 Institute of Nanotechnology (INT), Karlsruhe Institute of Technology (KIT), PO Box 3640, 76021 Karlsruhe, Germany; 4 Centre for Organismal Studies (COS), Heidelberg University, Im Neuenheimer Feld 230, 69120 Heidelberg, Germany; 5 Department of Physics, University of Illinois at Urbana−Champaign, Urbana, IL 61801, USA

**Keywords:** Membrane architecture, Cell biology

## Abstract

Repair of lesions in the plasma membrane is key to sustaining cellular homeostasis. Cells maintain cytoplasmic as well as membrane-bound stores of repair proteins that can rapidly precipitate at the site of membrane lesions. However, little is known about the origins of lipids and proteins for resealing and repair of the plasma membrane. Here we study the dynamics of caveolar proteins after laser-induced lesioning of plasma membranes of mammalian C2C12 tissue culture cells and muscle cells of intact zebrafish embryos. Single-molecule diffusivity measurements indicate that caveolar clusters break up into smaller entities after wounding. Unlike Annexins and Dysferlin, caveolar proteins do not accumulate at the lesion patch. In caveolae-depleted *cavin1a* knockout zebrafish embryos, lesion patch formation is impaired, and injured cells show reduced survival. Our data suggest that caveolae disassembly releases surplus plasma membrane near the lesion to facilitate membrane repair after initial patch formation for emergency sealing.

## Highlights

• Caveolae are involved in the cell’s response to local membrane damage
• Caveolar proteins do not accumulate in the early response (lesion plug formation)
• Caveolar clusters disassemble near the lesion site
• The presence of caveolae fosters cell survival after wounding

## Graphical abstract

**Figure:**
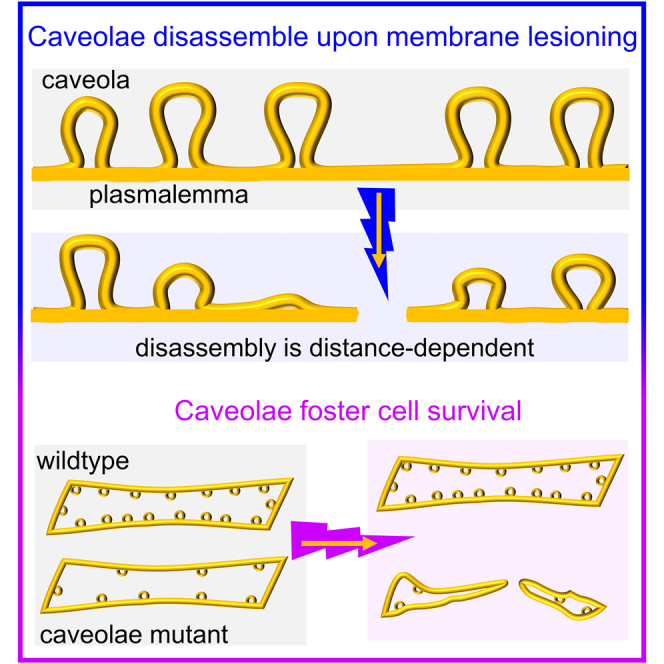

## Introduction

Maintaining concentration gradients across the plasma membrane is essential for cellular homeostasis. Therefore, muscle (and other types of) cells are endowed with effective repair systems, enabling them to quickly seal small lesions in the plasma membrane occurring under normal physiological activity. In recent years, our understanding of the molecular mechanisms underlying repair of the plasma membrane has been advanced considerably.^1^^,^^2^^,^^3^^,^^4^ Local influx of Ca^2+^ ions from the extracellular compartment triggers the accumulation of a number of cytoplasmic and membrane-bound proteins at the site of lesion. Within seconds, proteins and lipids, including phosphatidylserine (PS) and cholesterol, aggregate and form a plug that provisionally seals the membrane hole.^2^^,^^5^ In a subsequent, less well-understood step, the plasma membrane is reconstituted beneath the plug. As a result, the plug is displaced to the extracellular space and endocytosed by macrophages.^5^

In earlier work, we employed the transparent zebrafish embryo and murine C2C12 cells as model systems to analyze muscle membrane repair in real time and in a native tissue context.^5^^,^^6^ Using laser irradiation to locally inflict membrane lesions of 2–4 μm diameter in individual cells expressing fluorescently tagged repair proteins, we followed the assembly of the repair plug in real time by fluorescence microscopy^5^^,^^6^ and electron microscopy.^7^ Repair proteins were found to congregate at the membrane lesion in a specific sequence: While the membrane protein Dysferlin (Dysf) and cytoplasmic AnnexinA6 started to accumulate within 30 s after lesioning, other Annexins such as Annexin2a arrived more slowly.^6^ In addition, PS and cholesterol were rapidly enriched at the lesion, indistinguishable in their kinetics from Dysf.^5^ Consequently, the repair patch was decorated with PS on its extracellular side, which in turn invites macrophages to endocytose the lesion plug.^5^

In this scenario, a key question is how the essential repair materials, lipids and proteins, are held available, so that they can swiftly be supplied to the wound. Membrane rupture and the ensuing local influx of Ca^2+^ ions lead to activation of Annexins to form insoluble complexes at the lesioning site.^6^^,^^8^^,^^9^^,^^10^ Annexins are known to be highly abundant in the cytoplasm,^6^^,^^10^ which ensures their immediate availability at leakage sites throughout the entire sarcolemma. Less clear, however, is the source of (membrane-associated) Dysf and lipids accumulating at the lesion plug. Cytoplasmic vesicles have been proposed as carriers delivering Dysf as well as lipids such as PS and cholesterol to the repair plug.^3^^,^^11^^,^^12^^,^^13^^,^^14^^,^^15^ However, in a systematic assessment of vesicular markers, we did not find any evidence of lysosomal, endocytic, or exocytic vesicles accumulating in the repair patch.^6^ Under the electron microscope, repair patches had an amorphous structure, only occasionally showing inclusions of vesicles.^7^ Thus, we hypothesized that the intact plasma membrane surrounding the lesion could be the supplier of lipids and Dysf.^5^^,^^6^ Several cell types including skeletal muscle, cardiomyocytes, fibroblasts, and endothelial cells show an abundance of caveolae, 50–80 nm diameter invaginations of the plasma membrane decorated with a characteristic set of membrane proteins including Caveolins and Cavins as scaffold structures,^16^^,^^17^^,^^18^^,^^19^^,^^20^ and Pacsin2, Dynamin2, and EHD2 influencing the caveola internalization dynamics.^21^^,^^22^^,^^23^ Exerting systemic mechanical or hypo-osmotic stress causes the caveolar pouches to flatten and disassemble, releasing their membrane material to the bulk of the plasma membrane.^24^^,^^25^^,^^26^^,^^27^ Thus, caveolae may be a potential source of lipids for lesion plug formation and, possibly, also for plasma membrane reconstitution after lesioning. In support of such a scenario, we note that caveolae are rich in PS and cholesterol,^17^ which rapidly accumulate in the repair patch.^5^ Moreover, Dysf was shown to interact with Caveolins.^28^

Besides disassembly in response to mechanical stress, caveolae can also bud off the plasma membrane to form intracellular vesicles for transcytosis and other types of membrane trafficking.^17^^,^^29^ Lesions inflicted by pore-forming agents^30^ or treatment with glass beads^31^ in tissue culture cells resulted in the endocytosis of caveolae and the formation of 80 nm-diameter vesicles positive for the caveolar membrane marker protein Caveolin1 (Cav1), suggesting that caveolae may internalize upon injury and supply lipids to the lesion in the form of small cytoplasmic vesicles. However, in our study of laser-induced lesions in muscle cells of zebrafish embryos using correlative light and electron microscopy, we did not find any such vesicles near the repair plug.^7^ There is a caveat in this observation, though, as caveolae as well as caveolar vesicles could have been destabilized during the freeze-substitution process.^7^ Moreover, wounds inflicted by a laser are larger than those induced mechanically by beads or by pore-forming agents. Taken together, a clear picture of the mechanistic involvement of caveolae in lesion repair has not yet emerged.

Here we have tested the hypothesis that membrane lesions trigger unfolding of caveolae, using both the zebrafish embryo and mouse C2C12 myoblasts as model systems. We provide evidence that caveolae unfold upon lesioning of muscle cells of intact zebrafish embryos and C2C12 cells, whereas endocytosis and vesicle accumulation in the lesion patch did not occur. Lesion plugs show minor structural variations in caveolae-deficient *cavin1a* (also denoted *ptrf1alpha*) knockout zebrafish embryos. They appear, however, to be fully competent to seal the membrane hole, suggesting that caveolae do not serve as reservoir for patch proteins and lipids. When assessed for survival after injury, *cavin1a-*deficient cells show a significantly reduced survival rate, suggesting that *cavin1a* and thus caveolae are supportive of membrane repair. Dissolution of the caveolar pouches can facilitate rapid expansion of the plasma membrane and provide additional membranous materials for the reconstitution of the plasma membrane underneath the lesion plug, thereby displacing the plug to the extracellular space.

## Results

### Caveolar proteins do not accumulate at the site of a membrane lesion

**Figure 1:**
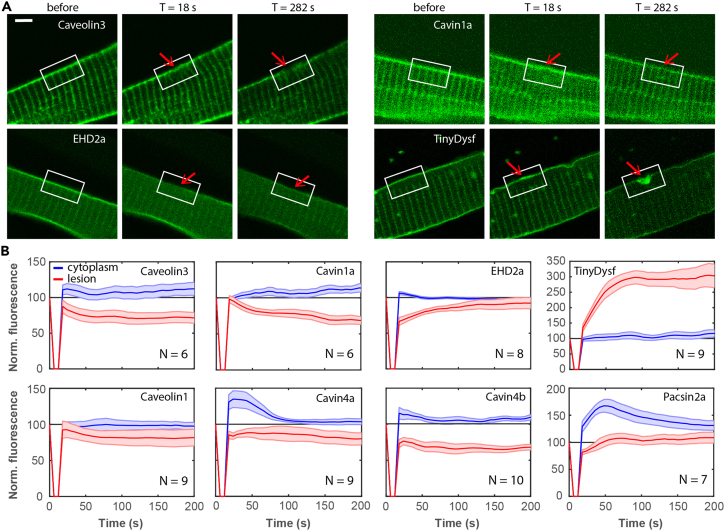
Caveolar proteins do not accumulate at the plasma membrane lesion site in zebrafish embryos (A) Caveolar proteins do not accumulate in the lesion patch. Fluorescence images of individual cells expressing Caveolin3-Clover, Clover-Cavin1a, EHD2a-Clover, and TinyDysf-Clover before and after laser-induced damage. The lesioning sites are marked by red arrows; white boxes surrounding the lesion sites mark regions with pronounced intensity changes. Scale bar (applies to all panels), 5 μm. (B) Caveolar proteins show distinct subcellular distributions after membrane lesioning. Time-dependent fluorescence of caveolar proteins fused to Clover at the lesion site (red) and in the cytoplasm (blue), respectively. Thick lines show averages over multiple cells (cell numbers, *N*, are given in each panel); shaded regions indicate standard errors of the mean. Fluorescence signals were set to 100 at *t* = 0.

Using confocal fluorescence microscopy, we aimed to assess the hypothesis that caveolar proteins contribute to the membrane lesion plug by observing changes in the subcellular distribution of caveolar proteins upon membrane injury. To this end, we generated fluorescent variants of several important zebrafish caveolar proteins by fusing the cDNA of the green fluorescent protein Clover^32^ in-frame at the N-terminal or C-terminal end (Tables S1 and S2). Upon injection of the *unc45b* promoter-driven DNA expression constructs^33^ into 1- to 2-cell stage zebrafish embryos, we exploited the resulting mosaic expression of the constructs in single cells of the somitic musculature of three-day-old zebrafish embryos. Small membrane lesions were inflicted in these cells by laser irradiation.^5^^,^^6^ Using time-lapse imaging in combination with quantitative analysis, we measured the temporal evolution of the fluorescence emission intensity and, thereby, the protein abundance at the site of the lesion before and for up to 282 s after membrane wounding by taking one image every 6 s. Representative examples are shown in Figure 1A. We did not find accumulation of caveolar proteins; instead, the signals of Caveolin1, Caveolin3, Cavin1a, and Cavin4a/b decreased by ∼25%, and those of Pacsin2a and EHD2a stayed roughly constant (Figure 1B). Apparently, caveolar proteins do not contribute directly to the repair patch. Only the signal of the Dysf reporter construct WRRFK-TM-Clover (denoted superminiDysf^34^ or, simpler, TinyDysf) rapidly increased at the wound, as previously reported.^5^

To probe for a possible relocation of caveolar proteins from the sarcolemma to the cytoplasm, we determined the fluorescence intensity of the fusion constructs in the cytoplasm between two Z-lines 2–4 μm away from the lesion spot (for details, see STAR Methods section) as a function of time (Figure 1B). After lesioning, the fluorescence and thus the concentration of Pacsin2a and Cavin4a in the cytosol increased by ∼75% and ∼40% within ∼50 s and ∼25 s, respectively, and then decreased again. While Pacsin2a was still ∼1.2-fold enhanced in the cytoplasm after 200 s, the concentration of Cavin4a reached the pre-wounding level after ∼100 s. The concentrations of the other proteins in the cytosol increased by only 10%–20%. In summary, the observed intensity changes upon membrane damage do not provide any evidence of caveolar proteins accumulating in the lesion patch.

**Figure 2:**
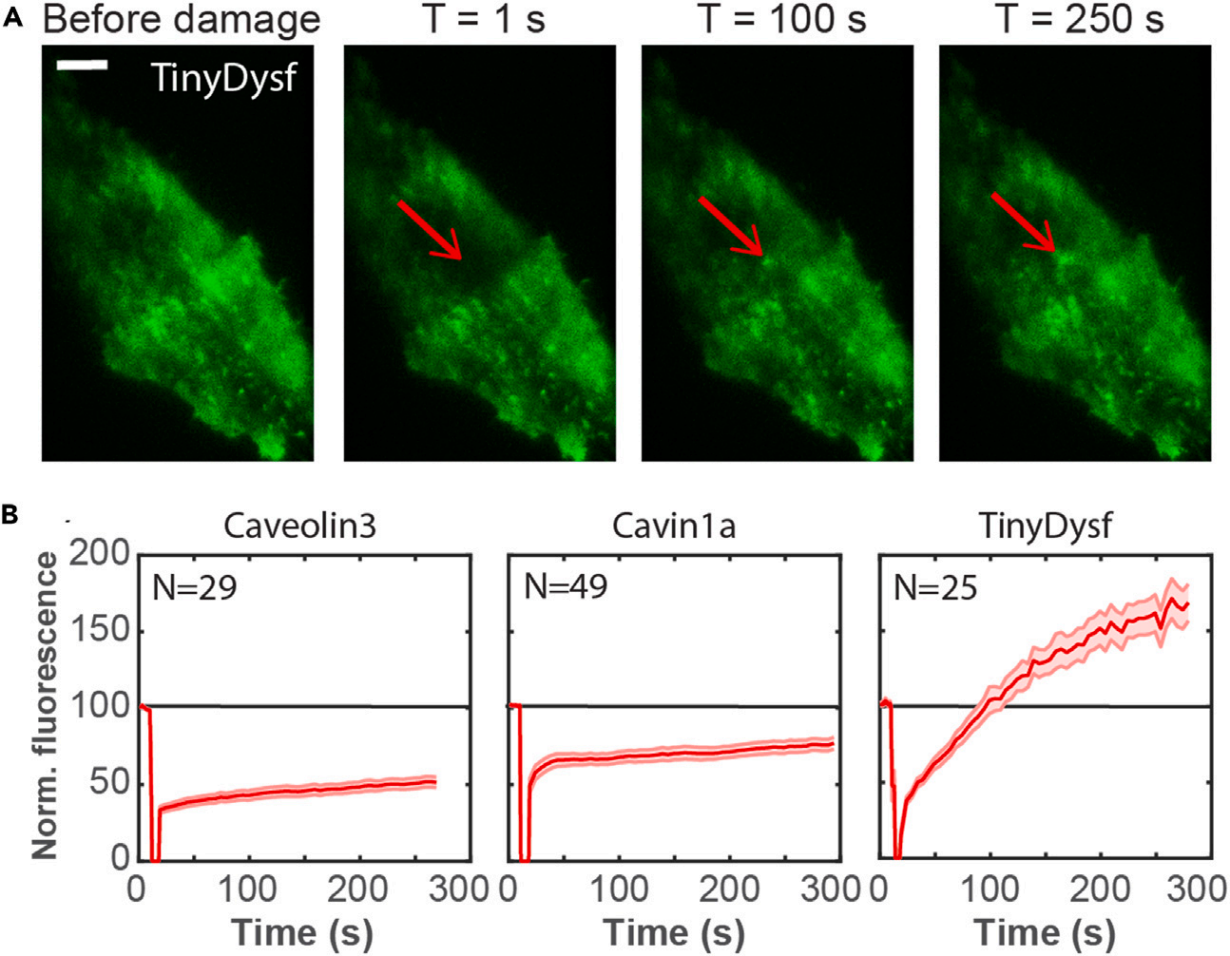
Caveolar proteins do not accumulate near a lesion inflicted in the C2C12 cell membrane (A) Representative images of the accumulation of TinyDysf-Clover at the site of a laser-induced lesion (marked by red arrow). Scale bar, 5 μm. (B) Time-dependent fluorescence of Caveolin3-mGarnet2, Cavin1a-mGarnet2, and TinyDysf-Clover at the lesion site. Thick lines show averages over multiple cells (cell numbers, *N*, are given in each panel); shaded regions indicate standard errors of the mean. Fluorescence signals were set to 100 at *t* = 0.

Next, we assessed whether caveolar proteins behave in a similar manner in mouse muscle myoblast C2C12 cells. We transiently transfected the cells with either *TinyDysf:Clover* or *Caveolin3:mGarnet2, Cavin1a:mGarnet2* to express fusion proteins with the far-red fluorescent protein mGarnet2.^35^ Then, we collected time-lapse images of the cells before and after inflicting a plasma membrane lesion by using a focused 405-nm laser beam. From these images, we again quantitatively analyzed the fluorescence intensity at the lesion site as a function of time. TinyDysf was found to strongly accumulate at the site of lesion (Figure 2), whereas the amount of Caveolin3 at the membrane dropped to ∼40% of the initial value and eventually recovered to ∼55% and that of Cavin1a dropped to ∼50% and eventually recovered to ∼80% (Figure 2). We conclude that the immediate response of TinyDysf and key caveolar proteins to laser-induced membrane damage in mammalian C2C12 cells is similar to that in zebrafish embryos.

### Caveolae disassemble near a lesion

**Figure 3:**
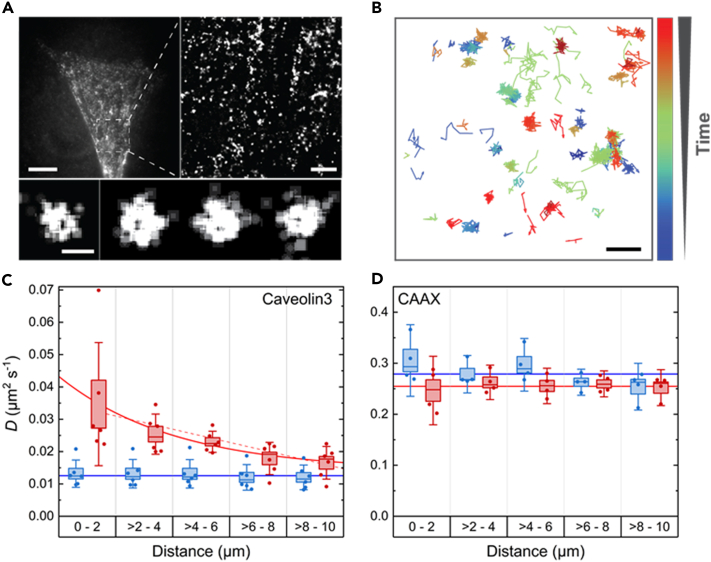
Diffusional dynamics of Caveolin3 and TinyDysf within C2C12 cell membranes before and after laser damage (A) Conventional epifluorescence image of a C2C12 cell transfected with Caveolin3-mEosFP*thermo* (upper left). Individual dots correspond to caveolar structures (scale bar, 10 μm). Close-up of the region marked by the white square, obtained by SMLM analysis (upper right, scale bar, 2 μm). Representative examples of mature caveolae (bottom, scale bar, 100 nm). (B) Example trajectories showing the movement of individual Caveolin3-mEosFP*thermo* proteins. Color bar, time in the image sequence (0–9 s); scale bar, 500 nm. (C and D) Average diffusion coefficients, *D*, of Caveolin3-mEosFP*thermo* and CAAX-mEosFP*thermo* before (blue) and within 2 min after (red) lesioning as a function of the distance to the lesion site. Each data point corresponds to *D* log-averaged over all trajectories recorded on at least five cells on a single day (in total six and four days for Caveolin3 and CAAX, respectively). Boxes indicate mean ± SD, the median is given by the line, and whiskers mark the 95% confidence level. Solid lines indicate the average over the data points shown in corresponding colors, except for the data after lesioning in panel C, which were fitted with an exponential, $D\left(r\right)=D_{0}\;\exp\left[-r/r_{0}\right]+D_{\infty }$, yielding $D_{0}$ = 0.029 μm^2^ s^−1^, $r_{0}$ = 3.7 μm and $D_{\infty }$ = 0.014 μm^2^ s^−1^ with a coefficient of determination, *R*^2^ = 0.98. A line fit, $D\left(r\right)=mr+D_{0}$, is also included as a dashed line, yielding slope m = −2.3 × 10^−3^ μm s^−1^ and offset ${\;D}_{0}$ = 0.035 μm^2^ s^−1^, with *R*^2^ = 0.91.

To examine if caveolae disassemble upon membrane damage, we probed the mobility of the muscle-specific Caveolin3 in the plasma membrane before and after damage. Nixon et al.^36^ have shown that caveolae are abundant along the muscle cell sarcolemma of two-day-old zebrafish embryos and that Caveolin3 colocalizes with these structures. Therefore, we studied C2C12 cells expressing Caveolin3-mEosFP*thermo* by single-molecule localization microscopy (SMLM), using a widefield microscope with total internal reflection fluorescence (TIRF) excitation.^16^^,^^37^ The fluorescent protein marker mEosFP*thermo* can be photoconverted from a green-emitting to a red-emitting form by 405-nm light irradiation.^38^^,^^39^^,^^40^ With sparse, stochastic photoconversion, we ensured that only a small fraction of mEosFP*thermo* labels was active in the red color channel in each frame, so that we could localize them individually with high sensitivity and precision. From sequences of typically 10,000 camera frames, super-resolution images were reconstructed showing individual caveolar structures in the plasma membranes (Figure 3A).

From mEosFP*thermo* localizations in consecutive SMLM images, we extracted two-dimensional time trajectories of individual fluorophores diffusing in the plasma membrane (Figure 3B). Although the trajectories are short (comprising only 7–8 frames on average, see STAR Methods and Figures S1B and S3B) due to the unavoidable fluorophore photodestruction, they nevertheless allow us to assess the diffusional dynamics of the fluorescence markers in the plasma membrane. To inquire if caveolae disassembly occurs only in membrane regions near the lesion, we tracked individual Caveolin3-mEosFP*thermo* molecules within 2 μm-wide annular regions around the site of lesion, with outer radii of 2, 4, 6, 8, and 10 μm, and performed mean square displacement (MSD) analyses (STAR Methods) of these trajectories. The resultant diffusion coefficients, *D* (averaged on a logarithmic scale), are reported in Figure 3C together with box-and-whiskers plots. Before membrane damage, the average diffusion coefficient, *D* = 0.013 ± 0.004 μm^2^s^‒1^ (mean ± standard deviation [SD] from measurements taken on different days), was obviously independent of the distance to the chosen site of lesion. After damage, we found *D* = 0.035 ± 0.018 μm^2^s^‒1^ in the central region. This value dropped to 0.016 ± 0.005 μm^2^s^‒1^ in the outermost ring, i.e., for trajectories 8–10 μm away from the lesion site. We have included exponential and linear fits in Figure 3C but, at this point, do not attempt to interpret the distance dependence with a physical model.

As a control, we performed the same analysis for the membrane-associated CAAX-mEosFP*thermo* construct, which carries a prenylation motif through which mEosFP*thermo* becomes attached to the plasma membrane (Figure 3D). For this lipid-anchored mEosFP*thermo*, the diffusivity was 10- to 30-fold greater than the one of the large membrane-associated Caveolin3-mEosFP*thermo* fusion. The changes in diffusivity (averaged over all five annuli) between pre-lesioning and post-lesioning were not significant, 0.28 ± 0.03 μm^2^s^‒1^ and 0.26 ± 0.03 μm^2^s^‒1^, and the distance dependence was small and insignificant. Therefore, the observed higher average mobility of Caveolin3-mEosFP*thermo* after membrane damage presumably reflects disassembly of caveolae close to the lesioning site rather than membrane perturbation (e.g., change in tension). Further support for this interpretation will be given in the next section.

### Membrane damage affects the diffusional dynamics of caveolar proteins

**Figure 4:**
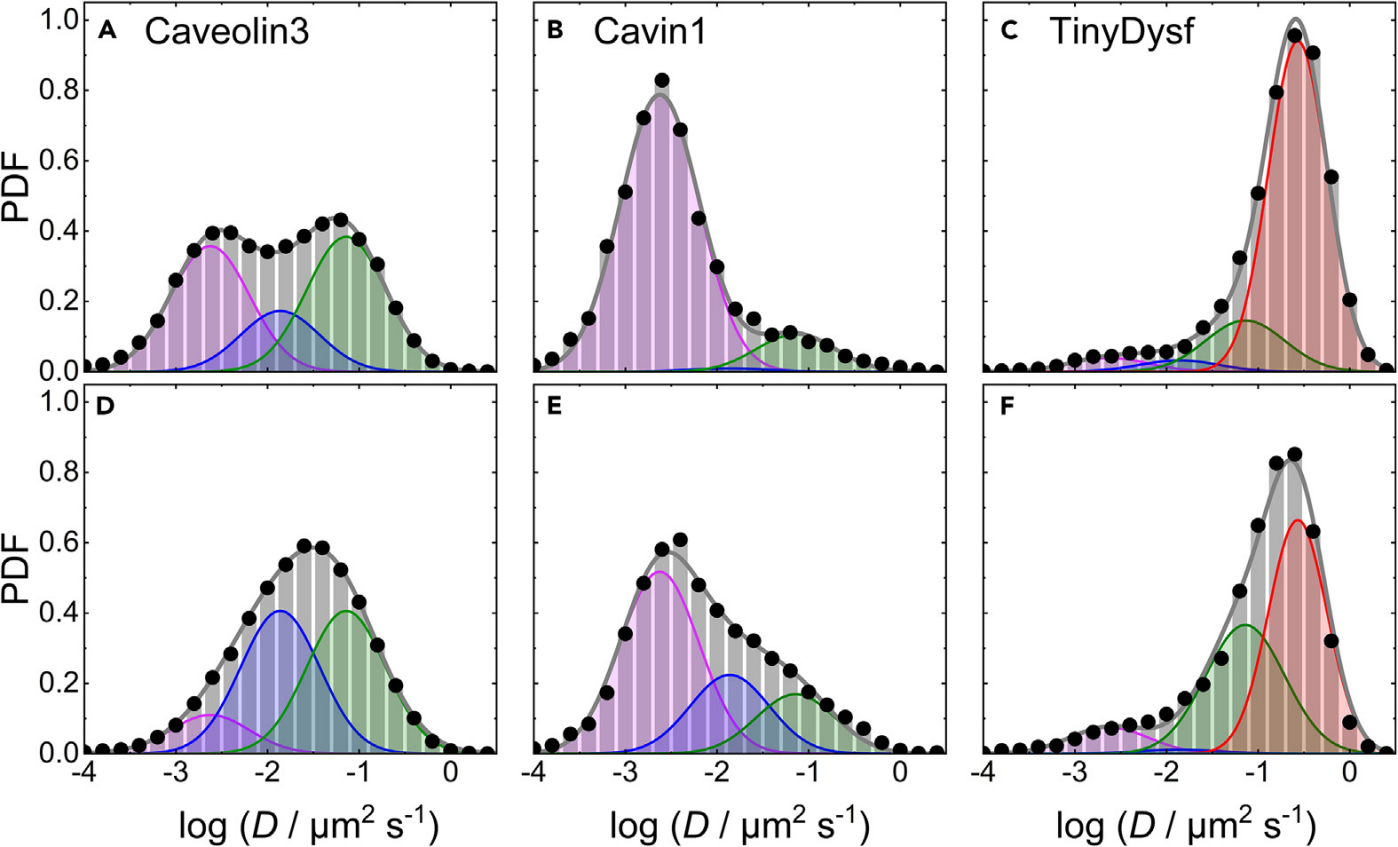
Area-normalized histograms (PDFs) of the number of mEosFP*thermo* fusion protein trajectories versus the diffusion coefficient, *D*, (on a logarithmic scale) Top (A–C) and bottom (D–F) rows, data taken before and after lesioning, respectively. The data were obtained by MSD analysis of trajectories extracted from TIRF image sequences of C2C12 cells transiently expressing mEosFP*thermo*-tagged (A, D) Cavin1, (B, E) Caveolin3, and (C, F) TinyDysf. Symbols and bars, data; gray line, sum of log-Gaussians fitted to the histograms; individual log-Gaussian distributions are included in magenta, blue, green, and red.

Next, we compared the diffusional dynamics of Caveolin3, Cavin1, and TinyDysf in C2C12 cells before and after membrane lesioning, using mEosFP*thermo* fusion constructs and transient transfection. After stochastic photoconversion, we tracked the diffusion of individual fusion proteins in the plasma membrane within a circular area of 4 μm radius centered on the lesion site before lesioning and during the first 2 min thereafter. For comparison, we also probed the diffusion of mEosFP*thermo* fusions with the PS sensor LactadherinC2 (LactC2), the GPI anchor (a lipid raft marker), and the prenylation motif CAAX. Using MSD analysis, we determined diffusion coefficients, *D*, from thousands (up to ∼73,000; for details, see Table S3) of single-molecule trajectories for each construct. These data were compiled in histograms of the numbers of trajectories with *D* values within certain intervals, using bins of constant width on a logarithmic scale. This approach is preferable to linear spacing because the trajectories are short due to photobleaching and, consequently, the uncertainties of the *D* values are large and distributed according to log-Gaussians (see Materials and Methods). The histograms were normalized to unit area to represent probability density functions (PDFs), quantifying the chance of finding a diffusion event with a particular value of $\tilde{D}=\log\;\left[D/\left(1\;\mu m^{2}s^{‒1}\right)\right]$ within a bin of width $\Delta \tilde{D}$ = 0.2. The resulting PDFs of Caveolin3 and Cavin1 are shown in Figure 4. For both proteins, already qualitative inspection of the PDFs shows a substantial loss of the leftmost and thus slowest-moving fraction in the spectrum of trajectories of diffusing particles upon lesioning.

For quantitative analysis, we fitted all four PDFs of Caveolin3 and Cavin1 with sums of three log-Gaussians corresponding to diffusing entities with clearly different mobility, as indicated by their markedly different center positions (log-averages), ${\tilde{D}}_{i}$ (Figure 4; Table S3). The widths of the three log-Gaussians, ${\tilde{\sigma }}_{i}$, reflect mainly the large uncertainty in $\tilde{D}$ determination from the short trajectories rather than dynamic heterogeneity. Therefore, the widths were kept identical and thus modeled as a single parameter. Moreover, we treated the parameters ${\tilde{D}}_{i}$ and ${\tilde{\sigma }}_{i}$ of the log-Gaussians as shared (joint) parameters for simultaneous (global) fitting of datasets from multiple experiments, which further reduces the number of free parameters and thus strengthens the significance of our analysis. This approach is motivated by the physical nature of caveolar structures. Cavin1 is an adaptor protein and a crucial component of mature caveolae, stabilizing their invaginated structures in the plasma membrane.^41^^,^^42^ Notably, it does not bind to (non-caveolar) Caveolin oligomers in the plasma membrane or at the Golgi. Thus, we expect the same diffusional signature for the two proteins in proper caveolar structures, as Cavin1 co-migrates together with Caveolin3. This approach is further supported by the finding that colocalization between Caveolin3 and Cavin1 in HeLa cell membranes is reduced (but not abolished) upon flattening of caveolae under mechanical stress exerted by hypo-osmotic shock.^24^ All fit parameters are compiled in Table S3; the fractions of the different populations obtained from the fits as well as exemplary diffusional trajectories are depicted in Figure S1.

The histograms of Caveolin3 (Figures 4A and 4D) display a broad range of diffusion coefficients and can be successfully fitted with three log-Gaussians, representing Caveolin3 clusters with widely different mobilities. Notably, our recent SMLM studies of caveolar structures have painted a highly dynamic picture, with incessant growth and decay of Caveolin scaffolds of widely different sizes, resembling the processes observed upon clathrin-coated pit formation.^16^ The slowest-diffusing log-Gaussian population 1, with center position, ${\tilde{D}}_{1}=‒2.63$ (i.e., *D*_1_ = 2.3 × 10^−3^ μm^2^s^−1^), and SD, ${\tilde{\sigma }}_{1}=0.43$, is practically immobile on our experimental times (trajectory durations) of up to a few hundred milliseconds (Figures 4A and 4D). Thus, we assign population 1 to mature caveolae, as they are large structures with low mobility in the plasma membrane, restrained by their interaction with the cortical actin cytoskeleton.^43^^,^^44^ The next, faster-diffusing population 2 is represented by a log-Gaussian with center position, ${\tilde{D}}_{2}=‒1.86$, and SD, ${\tilde{\sigma }}_{2}=0.43$ (Figures 4A and 4D). We assign this population to larger Caveolin3 assemblies with substantially higher freedom to move, including entire caveolae untethered from the cytoskeleton^22^ as well as larger non-caveolar assemblies. Notably, our *D*_2_ value agrees well with diffusivities of caveolae in HeLa cells after treatment with cytochalasin D (1 × 10^−2^ μm^2^s^−1^).^44^ Further details will be discussed in the following. A third log-Gaussian, with center position, ${\tilde{D}}_{3}=‒1.14$, and SD, ${\tilde{\sigma }}_{3}=0.43$, represents smaller diffusing entities, including small clusters and individual Caveolin3 protomers. Indeed, the mean diffusivity associated with population 3, ∼0.1 μm^2^ s^−1^, is in the range found for membrane receptors.^45^^,^^46^ Hirama et al.^47^ studied Caveolin1 diffusion using standard TIRF microscopy and arrived at very similar results as regards the diffusivity of the first two populations. They did not report a third, faster-diffusing population, however, presumably due to insufficient brightness of these entities for detection by conventional TIRF microscopy. Membrane lesioning caused a pronounced decrease of population 1 (immobile caveolae) from 39% to 12%. As the fastest Caveolin3 population was only slightly increased (Figures 4A and 4D; Table S3), most of the molecules lost from population 1 appeared in population 2 (Figures 4A and 4D). Thus, a large part of Caveolin3 acquires mobility upon lesioning.

The histograms of Cavin1 (Figures 4B and 4E) are also well described by our global fit with three log-Gaussians, as expected for Cavin1 bound to and co-migrating with Caveolin3. Before membrane damage, the immobile population 1 was predominant (87%) in the PDF, population 2 was non-existent within the error, and the fast-diffusing population 3 was small (Figure 4B). This is in accord with earlier observations, showing that Cavins either associate with mature caveolae (but not with Caveolin clusters in general) or bind to the plasma membrane as small clusters outside of defined membrane domains.^41^ Membrane damage caused population 1 to markedly decrease to 57% (Figures 4B and 4E). A substantial fraction (25%) appeared in population 2, indicating mobilization of caveolae, presumably by untethering from the cytoskeleton. The remaining part was found in population 3, implying disassembly of caveolae and formation of smaller Cavin clusters. Notably, the Cavin1 PDFs from before and after membrane lesioning are both intrinsically normalized distributions and thus do not provide information about the fraction of Cavin1 that dissociates from the plasma membrane due to wounding.

However, comparison of the Caveolin3 and Cavin1 PDFs allows further conclusions to be drawn. Before lesioning, there is a clear population 2 present for Caveolin3 (Figure 4A) but not for Cavin1 (Figure 4B). As Cavin1 only associates with mature caveolae, we conclude that this fraction comprises non-caveolar Caveolin3 clusters rather than mobilized caveolae, as reported earlier.^22^ They may arise due to overexpression of Caveolin3,^48^^,^^49^^,^^50^ and we note in addition that Caveolins have other functions unrelated to caveolae.^50^ Super-resolution fluorescence^51^ and cryo-electron microscopy^52^ studies have shown that Caveolin1 forms smaller complexes in the plasma membrane in the absence of Cavin1, which may further co-assemble into curved domains. In cells lacking Cavin1, Caveolin1 can generate membrane invaginations denoted dolines, which also flatten in response to increased membrane tension, like caveolae.^53^ Upon lesioning, population 2 increased markedly for both Caveolin3 and Cavin1, suggesting that caveolae became untethered from the cytoskeleton. Furthermore, the fractions in population 3 increased for both proteins, signaling the formation of smaller diffusing entities containing either Caveolin3 or Cavin1.

We analyzed exchanges between immobile and mobilized caveolae, as viewed through the dynamics of the two proteins. Without membrane damage, all Cavin1 caveolar structures were associated with the immobile population 1. Upon lesioning, this population dropped considerably, and 29% of this fraction appeared as mobilized caveolae (population 2). Likewise, all Caveolin3 caveolar structures were associated with the immobile population 1 without membrane damage, as population 2 of the Caveolin3 PDF (Figure 4A) corresponds to non-caveolar Caveolin3 due to the absence of population 2 for Cavin1 (Figure 4B). Upon lesioning, population 1 dropped by more than a factor of three, and essentially all lost caveolar Caveolin3 (69%) appeared in population 2, as there was only a small increase in population 3. Importantly, we cannot tell from the Caveolin3 data alone whether these population 2 molecules are associated with mobilized caveolae or non-caveolar structures. However, Caveolins and Cavins have been shown to occur in a fixed stoichiometric ratio in mature caveolae, with absolute molecule numbers estimated to be ∼150 and ∼50, respectively.^16^^,^^54^^,^^55^^,^^56^ Therefore, if membrane damage merely induces untethering of mature caveolae but leaves the overall structure intact, we expect population changes in a proportionate fashion for the two proteins. Our finding (Figure 4) that 29% of Cavin1 but 69% of Caveolin3 from population 1 (immobile caveolae) were rendered mobile upon membrane damage indicates that less than one-half of the mobilized Caveolin3 molecules ended up in untethered caveolae (with attached Cavin1); the majority was associated with non-caveolar structures (without attached Cavin1). This result makes good sense in light of the fact that Caveolins are integral membrane proteins that cannot leave the plasma membrane, whereas Cavins are peripheral membrane proteins that only associate with the properly curved caveolar structure. Upon membrane damage, they can translocate into the cytosol, leaving behind non-caveolar Caveolin3 clusters.

We note that this analysis hinges on the assumption that the stoichiometric ratio of Caveolin3 and Cavin1 is identical for immobile and mobilized caveolae, which appears reasonable, considering that the structure of the bulb should not markedly change if it is severed from the cytoskeleton. Consequently, our analysis yields a coexistence of caveolar and non-caveolar Caveolin3 clusters. If we drop this assumption, the observed population changes would be in line with a homogeneous population 2 of Caveolin3 clusters carrying less than one-fifth of the Cavin1 normally bound to mature caveolae. We would not assign such diffusing clusters to bona fide caveolae.

Taken together, our analysis reveals dramatic changes in the diffusional mobility of Caveolin3 and Cavin1 upon membrane damage. We found a marked decrease of the fraction of mature, essentially immobile caveolae, which is accompanied by an enhancement of other diffusing entities, which may include untethered caveolae, non-caveolar Caveolin3 structures, and smaller clusters of the two key caveolar scaffolding proteins.

For the SMLM experiments with TinyDysf, the mEosFP*thermo* marker was attached to its (extracellular) C-terminal end. As TinyDysf is much smaller than Caveolin3, we anticipate a larger diffusivity. Indeed, the histograms in Figures 4C and 4F show a huge, fast-diffusing population peaking at $D_{4}$ = 0.27 μm^2^ s^−1^ (Table S3), a value similar to those reported for other single-pass membrane proteins.^45^^,^^57^ However, populations with slower diffusion are also clearly apparent, indicating that TinyDysf can be part of larger structures in the membrane including immobile ones, e.g., Caveolin3 aggregates or even mature caveolae. Without further information at hand, we have fitted the histograms with the three log-Gaussians found for Caveolin3 and Cavin1, varying only the fractional weights but leaving positions and widths unchanged, plus an additional log-Gaussian to account for the fastest fraction corresponding to free diffusion (Figures 4C and 4F). This latter component (population 4) comprises 76% of the trajectories before lesioning. Afterward, this peak develops markedly more skew toward the lower-diffusivity side and shifts ($D_{4}$ = 0.19 μm^2^ s^−1^), which the fit routine takes into account by increasing population 3, assigned to small membrane-associated clusters. On a critical note, although two log-Gaussians are required to fit the asymmetric shape of the peak, this does not prove that there are indeed two distinguishable populations. Importantly, however, even without any fitting, it is obvious that the fraction of TinyDysf in more slowly moving structures increases upon lesioning. This finding may reflect the enrichment of TinyDysf near the wound seen in Figures 1 and 2. Anyhow, the lesioning-induced breakup of caveolae, which was inferred from the Caveolin3 and Cavin1 data (Figures 4A, 4B, 4D, 4E), does not mobilize TinyDysf and generate more of its fast-diffusing fraction. Therefore, caveolae do not appear to serve as reservoir structures that release TinyDysf after membrane wounding.

**Figure 5:**
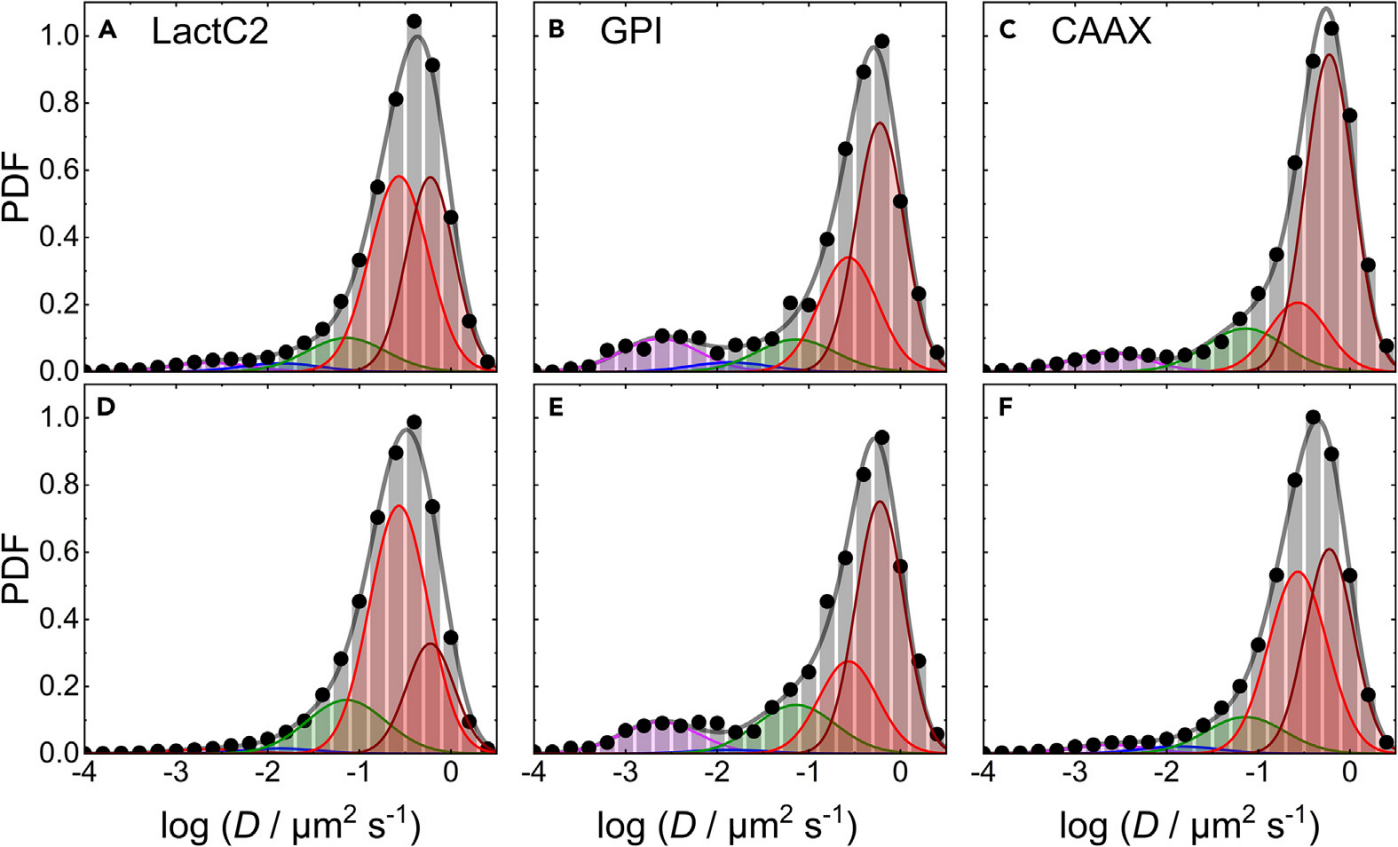
Area-normalized histograms (PDFs) of the number of mEosFP*thermo* fusion protein trajectories versus the diffusion coefficient, *D*, (on a logarithmic scale) Top (A–C) and bottom (D–F) rows, data taken before and after lesioning, respectively. The data were obtained by MSD analysis of trajectories extracted from TIRF image sequences of C2C12 cells transiently expressing mEosFP*thermo*-tagged (A, D) LactC2, (B, E) GPI anchor, and (C, F) CAAX motif. Symbols and bars, data; gray line, sum of log-Gaussians fitted to the histograms; individual log-Gaussian distributions are included in magenta, blue, green, red, and dark-red.

To further prove the stringency of our trajectory-based diffusional analysis, we performed studies of mEosFP*thermo* fusions with LactC2, GPI anchor, and CAAX (Figure 5). Like the TinyDysf data, all these PDFs show predominant, asymmetric bands peaking at even greater diffusivities than the one of TinyDysf (see overall peak positions in Table S3), as anticipated for constructs bound to the membrane via lipidic moieties, which are less bulky and thus more mobile than the transmembrane helix of TinyDysf. The fast-diffusing populations are accompanied by small, slowly diffusing and immobile fractions, as was already seen in the TinyDysf histograms. For fitting with multiple log-Gaussians, we kept the positions and widths of the four log-Gaussians from the TinyDysf fit (Figures 4C and 4F) and added a fifth log-Gaussian to better model the shape of the fastest-moving population. Its position and width were again shared among the three constructs. With a center position, ${\tilde{D}}_{5}=‒0.22$, this peak is close to that of population 4. Consequently, small shifts of the overall free-diffusion peak are captured by the fitting routine by adjustment of the relative weights of populations 4 and 5. Notably, the CAAX construct has the largest diffusivity, as expected for a single alkyl moiety inserted into the membrane (*D*_pk_ = 0.55 μm^2^ s^−1^), followed by the GPI anchor (*D*_pk_ = 0.48 μm^2^ s^−1^) with its phosphatidyl inositol lipid carrying two alkyl chains, and finally the LactC2 domain (*D*_pk_ = 0.43 μm^2^ s^−1^) with its two potential PS binding sites (all data before membrane damage, Table S3).^58^ For the GPI-anchored yellow-fluorescent protein (YFP), Parton and coworkers^59^ measured a diffusivity in the plasma membrane of mouse embryonic fibroblast cells identical to our *D*_pk_ value of 0.48 μm^2^ s^−1^, and similar values (∼0.5 μm^2^ s^−1^) were determined for other proteins GPI-anchored to the plasma membranes of PtK2^60^ and Chinese hamster ovary (CHO) cells.^61^ mEosFP^38^ fused to a membrane-targeting peptide^62^ and PS fused to a fluorescent dye^63^ also had average *D* values of 0.49 μm^2^ s^−1^

Upon membrane lesioning, the fast-diffusing populations of CAAX and LactC2 slow significantly, and their slowest fractions decrease (Figures 5A–5D, and 5F). Accordingly, they become liberated from larger aggregates but experience more resistance to diffusion in the damaged membrane. We note that the diffusional slowing is also seen for CAAX in Figure 3D. In contrast to LactC2 and CAAX, the PDFs of the GPI anchor (Figures 5B and 5E) display minimal perturbation of the diffusional dynamics by membrane damage, and GPI has the largest immobile fraction. These findings may be related to the fact that this glycolipid preferentially associates with larger membrane microdomains (lipid rafts).^64^ Interestingly, as for TinyDysf, there was practically no population 2 present for all three constructs (Figure 5, Table S3), implying that they tend not to associate with medium-sized caveolar structures (or membrane aggregates of similar size).

### Knockout of *cavin1* leads to lower numbers of caveolae in zebrafish muscle cells

Cavin1 is an essential component of caveolae and instrumental for their integrity in mammals.^18^^,^^20^ Zebrafish express two Cavin1 paralogs, Cavin1a and Cavin1b, in muscle and notochord cells, respectively.^65^ To assess the effect of Cavin1a on membrane repair, we generated a *cavin1a* knockout in zebrafish (*cavin1a*^*ka738/ka738*^, termed Δ*cavin1a*) by removing the entire coding sequence of *cavin1a* using the CRISPR/Cas9 method (Figure S2A).

**Figure 6:**
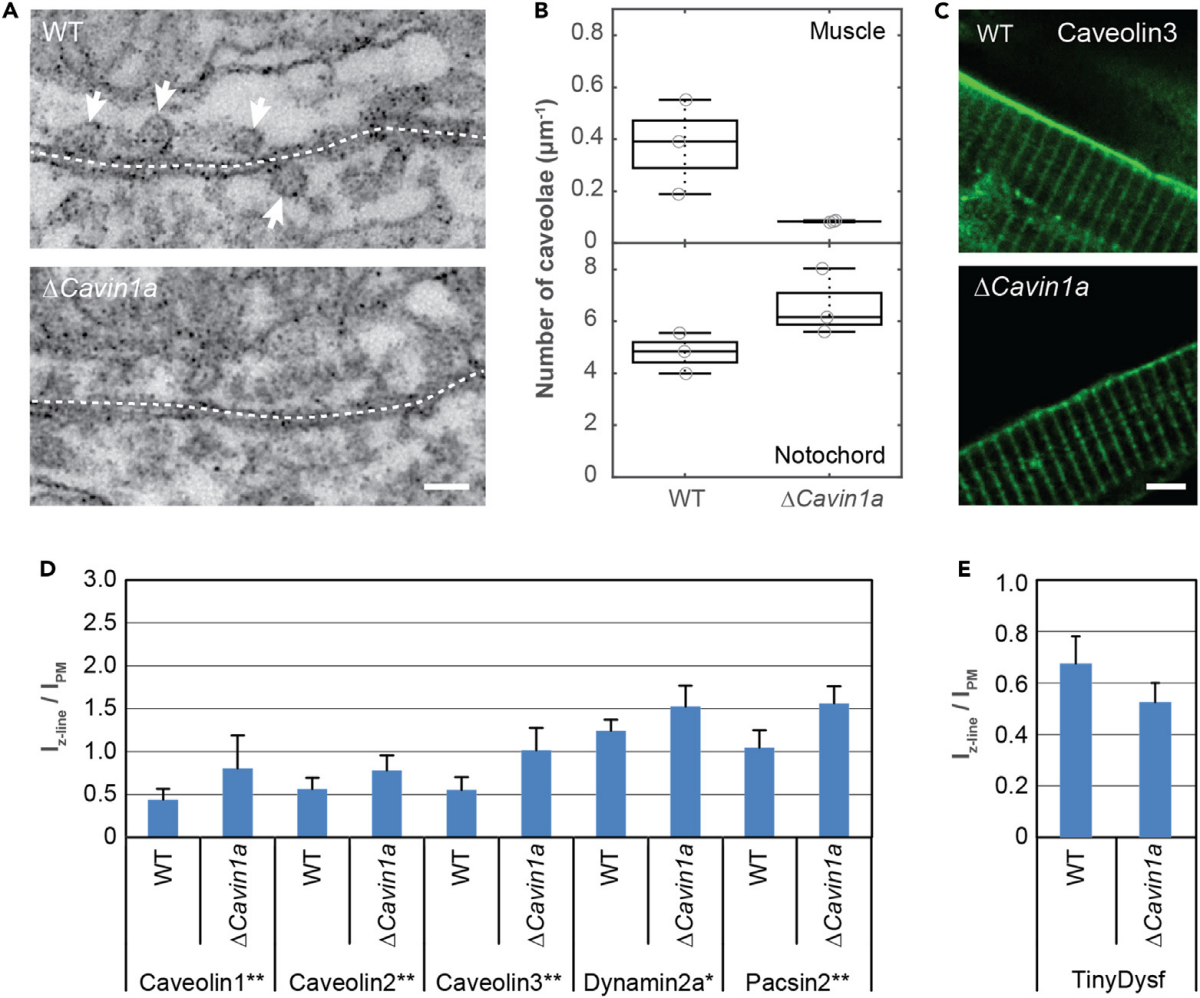
*Cavin1a* knockout zebrafish embryos show decreased abundance of caveolar proteins in the sarcolemma (A) EM images of slices of muscle tissue in wild-type (top) and Δ*cavin1a* zebrafish embryos (bottom). White arrows point to caveolar structures. Scale bar, 100 nm. (B) Number of caveolae in muscle and notochord cells of wild-type and Δ*cavin1a* zebrafish embryos. Each data point corresponds to the value determined for one muscle cell. (C) Representative fluorescence images of wild-type and Δ*cavin1a* strains injected with *caveolin3:Clover*. Scale bar, 5 μm. (D) Abundance of caveolar proteins tagged with Clover in the Z-lines, relative to the abundance in the sarcolemma. Error bars represent the SD. The significance was tested using the Bonferroni and Holm t test. (E) Abundance of TinyDysf-Clover in the Z-lines, relative to the abundance in the sarcolemma.

As a first step to characterizing the mutant, we imaged muscle cells of wild-type and Δ*cavin1a* embryos by transmission electron microscopy (Figures 6A and S2B) and quantified the number of caveolae in both strains (Figure 6B). Δ*cavin1a* embryos had significantly smaller numbers of caveolae in muscle cells but not in notochord cells (Figure 6B).

We next compared the subcellular distributions of various caveolar fusion proteins as well as TinyDysf in Δ*cavin1a* mutants and wild-type siblings. In wild-type cells, Caveolin3:Clover was mainly located in the sarcolemma; the signal from the Z-line was clearly lower (Figures 6C and S3A), as was earlier reported also by Hall et al.^66^ In mutant cells, the amount of Caveolin3:Clover on the sarcolemma was markedly lower than in wild-type embryos, whereas the amount in the Z-lines was comparable to that in wild-type cells (Figure 6C). Quantitative analysis showed that, in mutant cells, Caveolin3 was distributed evenly between sarcolemma and Z-lines (Figure S3B). In wild-type cells, Caveolin1 and -2 were also more prevalent in the sarcolemma; Pacsin2 and Dynamin2a were roughly evenly distributed between sarcolemma and Z-lines, indicative of the lacking caveolae in the Δ*cavin1a* mutant (Figure S3A). Like Caveolin3, the Caveolin1 and -2 fusion proteins were also distributed more evenly between sarcolemma and Z-lines of the mutant cells; Pacsin2 and Dynamin2 were more prevalent at the Z-lines (Figure S3B). In Figure 6D, we have plotted the abundance of these caveolar proteins in the Z-lines relative to the abundance in the sarcolemma. The ratio was always higher for the mutant cells, which likely reflects defective caveolar assembly. In contrast, TinyDysf was preferentially located at the sarcolemma in both wild-type and mutant cells (Figures S3A and S3B); there was no significant redistribution in the Δ*cavin1a* mutant (Figure 6E), implying that TinyDsyf is not involved in caveola formation.

### Lack of *cavin1a* does not impair function of the lesion patch but affects long-term cell survival

**Figure 7:**
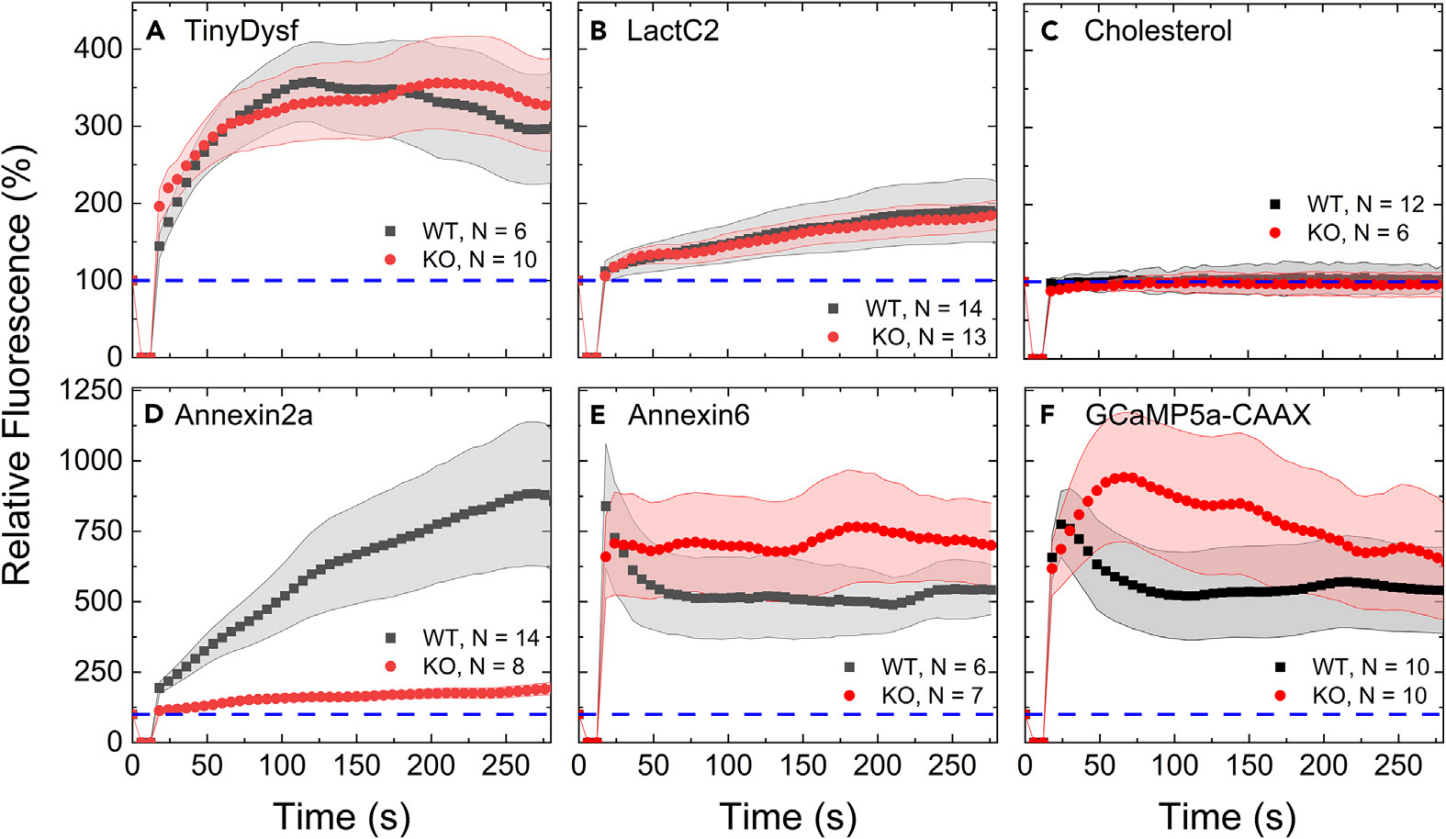
Δ*cavin1a* mutants have slightly abnormal lesion patches but appear functional Temporal development of the relative emission intensities of different proteins at the lesion site in the wild-type (black) and *cavin1a* knockout (red) strains, normalized to the fluorescence measured at the sarcolemma at the site right before wounding. (A) TinyDysf-Clover. (B) LactC2-EGFP. (C) BODIPY-Cholesterol. (D) Annexin2a-mOrange. (E) Annexin6-mOrange. (F) GCaMP5a-CAAX. Symbols show averages over multiple cells (cell numbers, *N*, are given in each panel); shaded regions indicate standard deviations. Fluorescence signals were set to 100% at *t* = 0 (before damage).

The mobilization of caveolar proteins near the membrane wound (see Figures 3 and 4) suggests a role of caveolar disassembly in response to membrane damage. We thus asked whether lesion patch formation as an immediate cellular response to a leaky membrane is disturbed in the Δ*cavin1a* mutant. We injected *tinyDysf* into zygotes of mutant and wild-type embryos, inflicted a lesion into an expressing somitic muscle cell in three-day-old embryos, and took confocal image sequences of the fluorescence intensity at the lesion site over time. TinyDysf accumulated at the lesion site in the mutant and in the wild type with essentially identical kinetics (Figure 7A). Similar behavior was found for the PS sensor LactC2 tagged with EGFP (Figure 7B) and the cholesterol sensor BODIPY-cholesterol^67^ (Figure 7C). In contrast, the Annexin2a-mOrange sensor displayed significantly less accumulation in the lesion patch in the mutant compared to the wild type (Figure 7D), whereas the Annexin6-mOrange sensor showed a slightly enhanced accumulation (Figure 7E). The different behavior of Annexin2a and 6 indicates a perturbed protein composition of the lesion patch of the Δ*cavin1a* mutant.

To test whether the formed lesion patches are functional and actually seal the membranes of Δ*cavin1a* mutants and wild-type siblings against Ca^2+^ ion influx, we employed the membrane-tethered Ca^2+^ sensor GCaMP5a:CAAX.^68^ The mutant cells showed a slightly increased presence of Ca^2+^ ions at the lesion site in comparison to wild-type cells (Figure 7F), suggesting a minor impairment of the sealing efficiency in the mutant. However, none of the injured cells died within the first 282 s, demonstrating that the lesion patch formed an effective barrier to the extracellular space. Thus, caveolae do not seem to play a role in establishing an effective patch, the immediate emergency response to wounding. Patch formation does not reconstitute the plasma membrane, however, and local disassembly of caveolae may be required to generate additional membrane surface to seal the plasma membrane behind the repair patch.

To test this hypothesis, we monitored the viability of injured cells in wild-type and Δ*cavin1a* backgrounds for 14 h post-injury. The rationale behind this experimental design is based on previous observations showing that cells (especially those with larger wounds) still died hours after inflicting a lesion.^5^ Death was presumably caused by mechanical constraints shearing off the lesion plugs or digestion of the plug by macrophages prior to full restoration of an intact plasma membrane. After inflicting a ∼1 μm diameter membrane lesion by near-infrared laser irradiation, cells were imaged 2, 6, 10, and 14 h after injury (Figures S4 and S5). This protocol resulted in 100% survival of injured wild-type cells (n = 9) within 14 h. In contrast, 9 mutant cells (n = 17) and thus 53% were dead 14 h after injury (p = 0.009, Fisher’s exact t test). Thus, the lack of *cavin1a* significantly reduced the survival probability of injured cells, supporting our notion that caveolae are involved in membrane repair. We can exclude a systemic 50% increase of cell death in the mutant, as such an obvious phenomenon cannot be overlooked even in a crude phenotypic analysis of the mutant. Moreover, we did not find apoptotic cells in uninjured cavin1a mutant muscle, which is another indication that cell death is associated with lesioning.

## Discussion

Membrane repair requires rapid mobilization of proteins and lipids to seal membrane ruptures and thus assure cell survival. This entails readily available stores of repair materials throughout the perimeter of the cell. We show here that plasma membrane lesioning triggers disassembly of caveolae as an immediate response. This process occurs in the vicinity of the lesion, causing a local expansion of the membrane. Systemic depletion of caveolae by deleting the coding region of *cavin1a* slightly disturbed formation of the lesion plug. However, the mutants still produced effective plugs that prevented cell death. This finding suggests that caveolae are not necessary for lesion patch formation as immediate first measure of the injured cell to seal the lesion. Remarkably, long-term cell survival was strongly reduced in Δ*cavin1a* mutant cells in comparison to injured cells in wild-type siblings. These observations support a model in which caveolae serve as reservoirs of membrane material for plasmalemma repair, a process that occurs on the cytosolic side of the lesion patch and eventually displaces it to the extracellular space.^5^ In summary, our data strongly suggest that disassembly of caveolae upon lesioning not only has a protective function, as has been proposed for mechanically stressed cells,^24^^,^^26^^,^^27^ but also may reflect active participation in membrane repair by making lipids stored in caveolae available for rapid membrane repair, as we will discuss below.

It is well established that mechanical stress of muscle cells causes systemic disassembly of caveolae, which protects the cell from membrane damage.^24^ It has yet remained unclear, however, whether such a response is also linked to local repair processes of membrane insults.^69^ Our data strongly argue for an involvement of caveolar disassembly in membrane repair. With diffusional trajectory analysis of SMLM image sequences of the plasma membrane, we showed that key caveolar proteins, Caveolin3 and Cavin1, are mobilized in response to laser-induced membrane injury, indicating a breakup of caveolae into smaller fragments (Figure 4). The change in mobility is greatest at the membrane wound and decays monotonically with distance (Figure 3). Our results fully support the notion that caveolae are locally dissolved at the lesion.

This observation raises the question how caveolar disassembly is triggered in such a spatially restricted manner. Mechanical stress may exert direct effects on caveolar structure and stability but also indirect effects through stress-sensitive molecular processes. A crucial component of caveolae are the BAR domain-containing Pacsin proteins, which can sense and generate membrane bends. Their membrane association is regulated by phosphorylation, mediated by Protein Kinase C (PKC).^70^ Phosphorylation of PACSIN2 by PKC in HeLa cells was shown to be induced by changes in membrane tension, caused by mechanical stimuli such as hypotonic or shear stress.^70^ As Ca^2+^ ions are among the various activators of PKC,^71^ it is conceivable that lesioning-induced Ca^2+^ influx activates PKC in a distance-dependent manner, with decreasing activation at greater distance to the lesion. This, in turn, leads to a gradient of Pacsin phosphorylation and, consequently, destabilization of caveolae in a similar gradient-like pattern around the membrane lesion. The aberrantly inflowing Ca^2+^ ions at the site of lesion also play crucial roles in the activation of other repair processes, e.g., the precipitation of Annexins in the repair patch,^6^^,^^72^^,^^73^^,^^74^^,^^75^ suggesting that Ca^2+^ ions orchestrate multiple emergency responses. Changes in the redox state of the cell due to membrane damage may yet be another trigger for caveolar disassembly, as caveolae were proposed to act as sensors for oxidative stress.^76^

At a membrane lesion, cells form a repair plug composed of proteins and lipids as an immediate measure to re-establish a tight boundary to the extracellular space.^2^^,^^5^^,^^6^ While the cytoplasm is a reservoir for globular proteins such as Annexins,^6^^,^^77^ integral membrane proteins such as Dysf are stored in the plasma membrane and in the T-tubules of muscle cells.^78^^,^^79^ It is still not clear where the lipidic material for membrane repair comes from. Lipids are present in the repair plug and are necessary for the subsequent resealing of the plasma membrane.

We have explored whether caveolae provide proteins and lipids for formation of the repair plug. We did not find accumulation of key caveolar proteins (Caveolin, Cavin) at the site of lesion in muscle cells of wild-type zebrafish embryos but, instead, observed relocation of some caveolar proteins (Pacsin2a, Cavin4a/b, see Figure 1B) to the cytoplasm. We can exclude cytoplasmic accumulation as an unspecific response to injury because Caveolin1 and -3, which are integral membrane proteins, stayed at the membrane (Figure 1B). Taken together, caveolar proteins do not relocate to the site of lesion to provide materials for repair.

However, Caveolin3 and Cavin1 showed markedly increased mobility at the membrane in response to injury (Figure 4), and the analysis revealed that caveolae break up into smaller diffusing entities. Note that this is a specific effect of membrane damage on the integrity of caveolae, as only minor effects were found for the membrane marker CAAX, the PS sensor LactC2, and the raft marker GPI (Figure 5). Disassembly (flattening) of caveolae is an effective means to locally expand the surface area of the plasma membrane and thus circumvents recruitment of extra lipid material to seal the hole. In the process, caveolae may first contribute specific lipids such as cholesterol and PS to the repair plug, which were previously shown to accumulate rapidly in the repair plug.^5^ However, we could not observe significant differences in the accumulation of fluorescent PS and cholesterol sensors at the lesion sites of the wild-type zebrafish strain (containing caveolae) and the Δ*cavin1a* mutant lacking caveolae (Figure 7), suggesting that caveolae do not serve as reservoirs for PS and cholesterol. It is, however, also possible that the metabolism and/or the localization of lipids is disturbed in the Δ*cavin1a* mutant, obscuring the role of caveolae as local reservoirs of PS and other lipids, or that LactC2 is not sensitive to PS trapped in caveolae, as was earlier reported for sensing cholesterol.^80^ Likewise, the Ca^2+^ sensor showed only minor differences between Δ*cavin1a* mutant and wild-type embryos at the lesion site, suggesting that the patch in the mutant effectively blocks influx of Ca^2+^ ions. In previous time-lapse studies of lesion patches that were sheared off by mechanical stress or removed by macrophages, unconstrained influx of Ca^2+^ ions led to rapid spreading across the entire cytosol, precipitation of Annexins on internal membrane structures, and cell death.^5^ We did not observe such fatal outcomes in our time-lapse studies of lesioned Δ*cavin1a* cells within the observation window of 282 s after lesioning, which supports our conclusion that the lesion plug is not functionally impaired in the mutant.

Earlier work reported that caveolae are internalized upon systemic injury of tissue culture cells with pore-forming agents.^31^ Our measurements suggest that caveolae do not undergo endocytosis, as we did not detect any bright, punctate features in the cytosol that could represent endocytic vesicles. We also did not find evidence of materials aggregating into larger intracellular structures, as discussed earlier.^69^ In our previous fluorescence and electron microscopy studies,^6^^,^^7^ we also did not detect any accumulation of vesicles in the lesion plug. Instead, with very few exceptions, the repair plug appeared as an amorphous structure under the electron microscope. These findings are in line with the data presented here, which demonstrate that caveolar proteins and thus caveolar vesicles do not accumulate in the lesion patch.

Instead, caveolae may serve as reservoirs of lipids for the second step in the cellular response to membrane injury, i.e., reconstruction of the plasma membrane. Local flattening of caveolae can markedly expand the surface of the plasma membrane, so that the plasma membrane bilayer can be reconstructed underneath the lesion plug. In this process, the repair plug is displaced to the extracellular space, where it is subsequently eliminated by macrophages attracted to the repair plug by PS.^5^^,^^7^ In agreement with this notion, we found reduced long-term survival in the Δ*cavin1a* mutant cells after wounding as compared to wild-type embryos. Since enhanced cell death was not observed for uninjured Δ*cavin1a* mutants (data not shown), it has to be correlated with prior injury of these cells. This finding suggests that reduced cell survival reflects an impaired capacity to repair membrane lesions in the mutant.

To assess the involvement of caveolae in membrane repair, we deleted the coding sequence of *cavin1a* in zebrafish to generate a knockout mutant. In view of the highly related *cavin1b* in the zebrafish genome, this knockout strategy bypasses complications by transcriptional adaptation^81^ and should thus represent a null phenotype. Electron microscopy revealed a strongly reduced number of caveolae in somitic muscle in mutant zebrafish embryos, but not in the notochord. As *cavin1a* is expressed exclusively in muscle, whereas the notochord expresses *cavin1b*,^65^^,^^82^^,^^83^ we conclude that *cavin1b* expression in the notochord accounts for the retention of caveolae in the notochord of Δ*cavin1a* mutants. This is supported by results from Hill et al.,^82^ who showed earlier that morpholino-modified antisense oligonucleotide (MO) knockdown of *cavin1b* reduces the caveolar density in the notochord of developing zebrafish embryos.

Furthermore, we observed that caveolar proteins were distributed differently between sarcolemma and Z-lines in wild-type and mutant muscle cells. In the Δ*cavin1a* mutant, the fraction of caveolar proteins in the sarcolemma was always lower than in wild-type cells. As ∼50% of the sarcolemmal area is occupied by caveolae,^65^ this finding agrees with the markedly lower number of caveolae at the sarcolemma observed by electron microscopy (Figure 6B). Moreover, it suggests that *cavin1a* and the formation of caveolae are required for quantitatively correct allocation of these proteins to the different membrane compartments in the uninjured cell. Accordingly, the distribution of the non-caveolar TinyDysf was not affected.

Parton and coworkers reported that the organization of T-tubules and sarcolemma was also disturbed in murine *cavin1−/−* mutant muscle cells.^65^^,^^84^ These cells resembled the phenotypes that we observed in somitic muscle cells of *cavin1a* mutant zebrafish embryos. Moreover, mutant murine muscle cells showed an increased sensitivity to mechanical stress, while *cavin1a* morphants showed sarcolemmal defects only after extensive mechanical stress.^65^^,^^84^ The latter finding is in contrast to the phenotypes that were found in our *cavin1a* deletion mutant and may reflect incomplete knockdown by the morpholino.

We^6^ and others^85^^,^^86^^,^^87^^,^^88^ reported earlier that Annexins contribute to the repair plug that forms within seconds after membrane damage. In the *cavin1a* mutants (Figure 7), the accumulation of Annexins is significantly different from that seen in wild-type cells, suggesting an impaired repair plug formation. After membrane lesioning, the overall concentration of the Annexin2 sensor increased only about 2-fold in the mutant cells but almost 10-fold in the wild-type cells.^5^^,^^6^ Moreover, the build-up was not complete within our observation time window of ∼300 s. In contrast, Annexin6 amassed within seconds at the lesion site of mutant cells to a level about 7-fold higher than before the damage, and the level in wild-type cells was about 5-fold increased. The underlying causes of these differences remain speculative. Both altered levels of Ca^2+^ influx and clearance could play a role. Annexins vary in their numbers of Ca^2+^ binding domains. The different kinetics of accumulation in the lesion plug may thus be a direct reflection of these different sensitivities to their activator Ca^2+^.^6^ We also note that Bittel et al.^89^ have recently shown that the accumulation of Annexin2 at the injured plasma membrane requires cholesterol, i.e., accumulation may be favored by the presence of cholesterol-rich caveolae in wild-type cells. In any case, these phenotypes of Δ*cavin1a* mutant embryos support the role of caveolae in membrane repair. The question if plasma membrane restoration in Δ*cavin1a* mutants is impaired requires further investigation with high-resolution microscopy including long-term, real-time imaging to follow the kinetics of membrane repair. Interpretation of the experimental data may not be straightforward, however, considering the defects in lipid metabolism of caveolae-depleted cells.^90^

To conclude, we studied processes subsequent to localized, laser-induced membrane lesioning in intact zebrafish embryos as well as in mammalian cells by using high-resolution confocal imaging as well as super-resolution SMLM. Quantitative analysis of SMLM image sequences revealed distributions of diffusivities of key caveolar proteins at the single-molecule level, showing local disassembly of caveolae in a spatially dependent fashion around the lesion. Moreover, the composition of the repair plug is altered in caveolae-depleted (Δ*cavin1a*) zebrafish mutants. Furthermore, we report a key involvement of caveolae in cell survival after wounding and suggest that caveolae serve as reservoirs for surplus materials for rapid repair of plasma membrane lesions.

### Limitations of the study

Our work clearly shows that caveolae do not serve as reservoirs for lesion plug materials but are nevertheless involved in plasma membrane repair, presumably in the restoration of a coherent plasma membrane. However, we could not directly demonstrate persistent defects in the plasma membrane of caveolae-depleted (Δ*cavin1a*) zebrafish mutants. The involvement of caveolae in the cellular response to membrane injury was clearly seen in our single-molecule dynamics studies, revealing a pronounced mobilization of Caveolin3 and Cavin1 upon laser-induced membrane lesioning. In the quantitative analysis of these data, discrete populations with markedly different dynamics have been identified. Their detailed interpretation in structural terms, however, relies on assumptions justified by results reported in the literature. Key open questions that remain to be tackled include the following: do caveolae indeed serve as reservoirs of lipids for wound closure and do they release membrane tension in the process? Direct visualization of plasma membrane repair, specifically the restoration of the membrane underneath the lesion patch, is extremely challenging and may require sophisticated correlative imaging techniques, which is beyond the scope of this work.

## STAR★Methods

### Key resources table

**Table:** 

REAGENT or RESOURCE	SOURCE	IDENTIFIER
**Chemicals, peptides, and recombinant proteins**		

DMEM	Thermo Fisher	11965092
Fetal bovine serum	Thermo Fisher	10270106
GeneArt Platinum Cas9 nuclease	Thermo Fisher	B25640, B25641
BODIPY-Cholesterol	Biomol GmbH	Cay24618-500
MESAB (tricaine, MS-222)	Sigma-Aldrich	E10521-10G

**Critical commercial assays**		

NEBuilder HiFi DNA Assembly Cloning Kit	New England Biolabs	E5520S
Ambion MEGAscript SP6 Kit	Thermo Fisher	AM1330

**Experimental models: Cell lines**		

C2C12 mouse C3H muscle myoblasts	Kind gift of Olivier Kassel	N/A

**Experimental models: Organisms/strains**		

AB_2_O_2_ wild type zebrafish	EZRC	N/A
*Cavin1a*^*ka738/ka738*^ zebrafisch mutants	Lab bred	N/A

**Oligonucleotides**		

Primers used for cloning	see Table S1	N/A
Gateway entry clones for constructing the expression vectors	see Table S2	N/A

**Recombinant DNA**		

cavin-1-mEGFP	Addgene	RRID: Addgene_27709
GPI_2xmCherry	Addgene	RRID: Addgene_127812

**Software and algorithms**		

Matlab	MathWorks	N/A
ImageJ	Github repository	N/A
Origin Pro 2022	Origin Lab	N/A
a-live PALM	available upon request	N/A

### Resource availability

#### Lead contact

• Further information and requests for resources and reagents should be directed to G. Ulrich Nienhaus (uli@uiuc.edu).

#### Materials availability

• Plasmids generated in this study are available upon request from the lead contact.

#### Data and code availability

• This paper does not report any original code.
• Any additional information required to reanalyze the data reported in this paper is available from the lead contact upon request.

### Experimental model and study participant details

#### Ethics statement

All experiments involving zebrafish conformed to the regulatory standards and guidelines of German animal protection regulations.

#### Zebrafish husbandry

Zebrafish were bred and maintained in accordance with German animal protection regulations (Regierungspräsidium Karlsruhe, Germany, AZ35-9185.81/G-137/10 and AZ35-9185.81/G-174/18).

### Method details

#### Zebrafish strains

AB_2_O_2_ wild type zebrafish (European Zebrafish Resource Centre EZRC, Karlsruhe) were used for all experiments. Zebrafish husbandry,^91^ breeding and experimentation were performed in accordance with German animal protection regulations (Regierungspräsidium Karlsruhe, Germany, AZ35-9185.81/G-137/10 and AZ35-9185.81/G-174/18).

#### Expression plasmids and sensors

Cloning of sensor and protein plasmids for expression in zebrafish embryos was carried out following standard procedures (supplemental methods). In brief, cDNAs of zebrafish gene orthologues of mammalian caveolar proteins were amplified with primers containing appropriate cloning sites (Table S1) and subcloned. Then they were fused with fluorescent reporters and inserted into the muscle specific expression cassette containing the *unc45b* promoter.^33^ Expression plasmids of the phosphatidylserine sensor LactC2, i.e., the C2 domain from Lactadherin, fused with EGFP,^92^ and TinyDysf fused with Clover, were reported previously.^5^

For SMLM experiments in C2C12 cells, mouse Cavin1 and the plasma membrane localization signal GPI were tagged with mEosFP*thermo* by replacing mEGFP in the Cavin1-mEGFP fusion (addgene #27709, Addgene, Watertown, MA) and both mCherry FPs in GPI-2×mCherry (addgene, #127812) with mEosFP*thermo*, respectively, using the NEBuilder HiFi DNA Assembly Cloning Kit (New England Biolabs, Ipswich, MA).

##### Cloning

PCR primers for amplifying DNA fragments (Table S1) were ordered from a commercial supplier (Metabion, Planegg, Germany). PCR for fragments shorter than 700 bp was performed using GoTaq/GoTaq2 polymerase (Promega, Mannheim, Germany). Longer fragments were amplified under low error rate conditions using either Pfu polymerase (Promega) with a gradient-PCR protocol (50°C - 70°C annealing temperature) or standard PCR with Q5 High Fidelity Polymerase (NEB, Frankfurt am Main, Germany). The elongation time was optimized for the insert length, assuming extension rates of 1,000 bp/min for GoTaq, and 500 bp/min for Pfu and Q5 polymerases. PCR products and vectors were purified from an ethidium bromide/agarose gel by using a gel purification kit (Peqlab, Erlangen, Germany), further digested by restriction enzymes (NEB, Fermentas, Darmstadt, Germany) and again purified from an ethidium bromide/agarose gel. To remove self-ligation products, the vectors were treated with thermosensitive alkaline phosphatase (Fermentas) for 1 h at 37°C and gel purified. Ligation was performed at a molar vector:insert ratio of 1:3 at 16°C overnight with T4 DNA Ligase (Promega), followed by heat shock-mediated transformation using *E. coli* strains XL1-blue or TOP10 (Thermo Fisher Scientific, Dreieich, Germany).

Expression vectors for fluorescent protein (FP)-tagged proteins were constructed by the Tol2kit, utilizing the multisite Gateway system.^93^ In general, the FP-tagged proteins and the regulatory elements to drive their expression were cloned into the middle entry vector (pME-MCS #237) and the 5′ entry vector (p5E-MCS #228), respectively. All entry vectors (p5E-, pME- and p3E vectors) were transformed into XL1-Blue MRF’ competent *E. coli* cells, which were plated on LB + kanamycin plates.

For muscle specific expression of the FP-tagged proteins in zebrafish, the unc45b promoter (from -505 to -310 relative to the ATG of unc45b) was cloned into the 5′ entry vector (p5E-MCS #228) between the BamHI and SacII restriction sites. Proteins of interest were fused with a FP at their N- or C-termini via a linker sequence (three repeats of Gly-Gly-Gly-Gly-Ser). For C-terminal tagging, PCR amplified mEosFP*thermo*,^38^ Clover or mGarnet2^35^ was inserted into the pME-MCS vector between the SpeI and SacI restriction sites. For N-terminal tagging, mEosFP*thermo* without stop codon was inserted between the KpnI and XhoI sites of the same vector. The linker sequence was integrated into the primer for C-terminal tagging; for N-terminal tagging, it was inserted into the pME backbone between the XhoI and ClaI sites.

To prepare a membrane-bound calcium sensor, the plasmid unc45b:GCaMP5A (#300) was used as a template. The CAAX motif (KLNPPDESGPGCMSCKCVLS) was introduced at the C-terminal of the calcium sensor by PCR. After Dpn1 digest at 37°C for 1 h, the DNA was transformed into TOP10 *E. coli* using ampicillin as selection marker for amplification and purification. The purified DNA was injected into one-cell stage zebrafish embryos at 30 ng/μl for transient expression of the calcium sensor.

The sensor for phosphatidylserine was constructed as middle entry clone. Therefore, different FPs were fused at their C-termini with the LactAdherinC2 domain. EGFP and LactAdherinC2 were individually PCR-amplified and cloned into the pME-MCS vector between the ClaI and XhoI sites, yielding pME-EGFP-LactAdherinC2. For pME-mEosFP*thermo*-LactAdherinC2, mEosFP*thermo* was fused via the linker (see above) with the LactAdherinC2 domain. CAAX was amplified and fused N-terminally to mEosFP*thermo* between the ClaI/SpeI sites. Sequences of used proteins and sensor constructs were confirmed by Sanger sequencing (Microsynth AG, Balgach, Switzerland).

For the expression vector construction, the 5′ entry clone with desired promoter (unc45b or CMV), the middle entry clone with the fusion construct, and the 3′ entry clone p3E-polyA #302 were recombined into the destination vector pDestTol2pA2 #394 using the LR clonase II kit (Invitrogen, Darmstadt, Germany). The reaction was setup by using 20 fmol of each plasmid prepared by using the Qiagen Plasmid Midi kit (Qiagen, Hilden, Germany) and incubated overnight at room temperature. After proteinase K digestion of the recombinase (37°C for 15 min), Top10 *E. coli* cells (Invitrogen) were transformed by heat-shock (42°C for 90 s) and selected on LB-ampicillin plates.

BODIPY-Cholesterol was dissolved in DMSO at 10 mM. 2 ‒ 3 nl of the BODIPY-Cholesterol stock solution (2 ‒ 3 pmol) were injected into the yolk of zebrafish embryos at the 1 ‒ 2 cell stages.

#### Knock-down and knock-out

*Cavin1a*^*ka738/ka738*^ mutants were created by CRISPR/Cas9-mediated deletion following the method as described.^94^ Briefly, two short-guide RNAs (sgRNAs, see Table S1) were designed using the CHOPCHOP web tool^95^ to delete the 35.6 kb of the *cavin1a* locus (3:16976415-17011998, GRCz11). Both coding exons (exon1 and exon2) including the start-ATG and stop codon (TAA) were flanked by the two designed sgRNAs. The template DNAs were prepared by T4 DNA polymerase-mediated fill-in reaction of two annealed synthesized oligonucleotides: one for site-specificity (either exon1 or exon2 of *cavin1a*) and another for a constant Cas9-binding sequence (Table S1).^94^ Both sgRNAs were transcribed *in vitro* from the template DNAs and purified by ammonium acetate precipitation (Ambion MEGAscript SP6 Kit, Thermo Fisher Scientific, Dreieich, Germany). 1 μl of each of the two sgRNAs (100 ‒ 300 ng/μl) were mixed with 1 μl of Cas9 protein (GeneArt Platinum Cas9 nuclease, 1 μg/μl, Invitrogen Darmstadt, Germany) and co-injected into the one-cell stage wildtype embryos to establish founder generation fish. Adult founder generation fish were outcrossed and the obtained embryos were processed for HotSHOT genomic DNA preparation.^96^ Genotyping was performed by PCR using a set of primers (Table S1).

#### Real-time imaging of membrane repair in intact zebrafish embryos

Plasmids encoding reporters were injected into the yolk of 1–2 cell stage embryos. Sarcolemmal lesions were generated using three day-old embryos, which were immobilized on a microscope cover slide using 0.5% low melting point agarose supplemented with 0.02% MESAB. Embryos were imaged with a water dip-in 63x objective (HC APO L 63x/0,90 W U-V-I; Leica Microsystems, Wetzlar, Germany) on a Leica TCS SP2 confocal microscope operated through the Leica confocal software (LCS). All observations were made at room temperature. The sarcolemma was damaged with a Ti:Sapphire laser (Mai-Tai, Spectra-Physics, Mountain View, CA, USA) set to 822 nm with an average output power of 1.6 W, which was fine-adjusted by an electro-optical modulator with gain and offset values set to 26% and 65%, respectively. In order to inflict lesions in the sarcolemma, two-photon illumination was applied in a 512-pixel format at 400 Hz (∼1.4 μs pixel dwell time) with the beam-expander value of 1. Illumination was limited to a few square-micron region within a field of view of 7.44 x 7.44 μm^2^ (32× digital zoom). Sensor accumulation at the membrane lesion was measured by determining the fluorescence intensity at the lesion in at least 5 independent experiments in a time period from 6 ‒ 282 s after lesioning.

#### Cell survival assay

Three-dpf zebrafish embryos from a macrophage-labeled transgenic line *Tg(mpeg1.1:GFP)*^*ka101Tg/ka101Tg*^ and *cavin1a* ^*ka738/ka738*^ homozygous mutants also carrying *Tg(mpeg1.1:GFP)*^*ka101Tg/ka101Tg*^ were embed in 0.5% (w/v) low-melting agarose in E3 medium supplemented with 0.02% MESAB (tricaine, MS-222). Approximately 20 embryos from each genotype were arranged in lateral orientation in the central part of a 6-cm petri plastic dish and immersed in 10 ml of E3 medium supplemented with 0.02% MESAB. Membrane damage was introduced by using a SP8 DIVE two-photon confocal system (Leica Microsystems, Wetzlar, Germany), equipped with an up-right DM6 microscope with a motorized stage and a HC APO L 63x/0.90 W U-V-I objective lens. Two-photon damage was introduced on the muscle membrane within a region-of-interest (ROI) of 1 μm^2^ size. The laser setting (excitation at 822 nm, with the gain value adjusted to 25%) was kept constant. To visualize damaged fibers and macrophages, confocal reflection using a 488 nm laser as the incident light and GFP fluorescence emission, respectively, were sequentially acquired every 15 min for a z-range of 20 ‒ 30 μm (slice interval 1 μm) for 12 h starting at 2 h post-injury (hpi).

#### Electron microscopy

For ultrastructural analysis of caveolae, 3-day wildtype and *cavin1a*^*ka738/ka738*^ mutant embryos were fixed for 1 h at room temperature in 2.5% glutaraldehyde, 0.05% ruthenium red in 0.1 M PIPES buffer at pH 7.0. Then embryos were washed with 0.1 M PIPES and fixed for another 3 h at room temperature in 0.5% OsO_4_, 0.05% ruthenium red in 0.1 M PIPES buffer at pH 7.0. After brief washing with 0.1 M PIPES, embryos were gradually dehydrated by exposing them to a series of aqueous solutions with increasing fractions (v/v) of ethanol (25%, 50%, 70%, 90%, 95% and 100%) and finally transferred to 1,2-propylene oxide. The samples were then incubated sequentially for 1 h at room temperature in 1:2 and 2:1 EPON812:propylene oxide solution, followed by 1 h in EPON812. Embryos were transferred in a silicon mold and oriented for sagittal sections. Polymerization was run to completion by overnight incubation at 65°C. Ultra-thin sections (70 nm) were prepared with an ultra 45° diamond knife (Diatome, Nidau, Switzerland) installed on a Leica EM UC6 ultramicrotome. Sections were recovered onto EM-slot grids (2 × 1 mm^2^ G2500PD; Plano, Wetzlar, Germany) that were manually coated with formvar film. No post-staining with uranyl or lead salts was performed. Images were acquired on a transmission electron microscope (Zeiss EM910) operated at 80 kV with 20,000-fold magnification.

#### Cell culture

Mouse C3H muscle myoblasts (C2C12) were cultured in high glucose DMEM (11965092, ThermoFisher Scientific, Waltham, MA, USA) supplemented with 10% fetal bovine serum (10270106, ThermoFisher Scientific) at 37°C and 5% CO_2_. The confluency of the myoblasts was kept below 70%.

Round glass coverslips (diameter 24 mm, No. 1.5H, Marienfeld, Lauda-Königshofen, Germany) were pre-cleaned in a plasma cleaner for 1 h and then incubated in 2% Hellmanex™ III (Hellma, Müllheim, Germany) at room temperature overnight. Afterwards, the coverslips were washed six times with MilliQ water, and stored under sterile conditions (for up to two weeks).

For imaging, the pre-cleaned round coverslips were put into 6-well plates. The myoblasts (in 2 ml medium) were transferred onto the coverslips and allowed to adhere for 24 h. Then, the cells were transfected with 3 μg of plasmid using Lipofectamine 3000 (L300008, ThermoFisher Scientific). After 5 h, the cell medium was replaced by fresh medium. Myoblasts were imaged 24 h post transfection. During imaging, myoblasts were kept in an environmental chamber heated to 37°C, with the gas atmosphere supplemented with 5% CO_2_.

#### TIRF and SMLM - Microscope setup and imaging procedures

Samples were imaged with a home-built total internal reflection fluorescence (TIRF) microscope based on a Zeiss Axio Observer Z1 body (Zeiss, Jena, Germany), as described previously.^16^^,^^37^ For fluorescence excitation, the setup employs a 405-nm (Stradus 405-250, Vortran Laser Technology, Sacramento, CA, USA), a 473-nm (Gem 473, Laser Quantum, Konstanz, Germany) and a 561-nm (Gem 561, Laser Quantum, Konstanz, Germany) laser. The laser beams are switched and their power is adjusted with an acousto-optical tunable filter (AOTF, model AOTFnC-400.650, A-A Opto-Electronic, Orsay, France). For widefield illumination of a large region of interest (ca. 70 × 70 μm^2^) with uniform intensity, a 10× beam expander is used, and the laser beam is focused onto the center of the back aperture of the objective lens (Zeiss α Plan-Apochromat 63x/1.46 Oil Korr M27, Jena, Germany). For total internal reflection (TIR) excitation, the focus is shifted to the side of the objective back aperture. The fluorescence images are collected with an EMCCD camera (Ixon Ultra X-7759, Andor, Belfast, UK) cooled to –90°C.

For laser-induced membrane lesioning, a half-wave plate was inserted into the beam path directly after the AOTF, and the 405-nm laser beam was diverted into a separate path by a polarizing beam splitter. The beam was expanded 2.5× and an additional tube lens was used to focus the beam on the sample. The beam was directed back to the original path by using another polarizing beam splitter. To inflict the lesion, the laser beam (5 mW at the damage site) was focused onto the cell membrane for 5 s. To measure protein accumulation in the lesion spot, 20 frames (500 ms dwell time, frame rate 1 Hz) were recorded before damage. To measure the temporal evolution of the fluorescence, 60 frames (dwell time 500 ms, frame rate 0.2 Hz) were taken immediately after lesioning.

For single molecule localization microscopy (SMLM), green-to-red photoconversion of mEosFP*thermo* photoactivatable fluorescent proteins^40^^,^^97^ was (randomly) induced by continuous illumination with weak 405-nm laser light to ensure that there were only a few mEosFP*thermo* molecules in the red-emitting state in each camera frame, so that they could be localized individually by their emission upon 561-nm laser excitation. Optimal SMLM imaging conditions were found with 405-nm and 561-nm laser powers in the range of 0.02 – 0.03 W cm^‒2^ and 0.05 – 0.06 kW cm^‒2^, respectively. The fluorescence emission was filtered with a 607/70 (center/width) nm bandpass filter and a 561 nm long pass filter. Typically, we captured image sequences (movies) for 300 s by collecting 10,000 image frames with 30 ms dwell time each.

#### Fluorescence microscopy data analysis

##### Quantification of protein accumulation in the lesion spot

We have analyzed temporal changes of the emission intensity from fluorescently tagged caveolar proteins in confocal image sequences to quantify their relocation after membrane lesioning in different parts of zebrafish embryos and C2C12 cells. To this end, a MATLAB (MathWorks, Natick, MA) user interface was written that enables us to process the image intensity data near the lesion site as follows. In zebrafish embryos, we select (i) a region centered on the lesion to quantify the signal at the lesion, *I*_raw_(*t*), (ii) a region outside of the embryo to correct for background, *BG*, and (iii) a region inside the sample but far from the lesion site to correct for photobleaching, *BL* (all 20 × 20 pixels). To correct for sample drift or movements of the live samples, the center of the lesion site was determined manually in each frame, and the relative positions of the other regions with respect to the center of the lesion spot were kept constant. In C2C12 cells, we selected a circular region on the membrane (diameter ∼1.5 μm) around the lesion site to quantify *I*_raw_(*t*). The fluorescence intensity in this region in the first frame captured after laser-induced damage was taken as BG. In zebrafish embryos and C2C12 cells, the emission intensities of all pixels belonging to the same region were averaged in each frame. The final intensity was calculated according to *I*(*t*) = (*I*_raw_(*t*) – *BG*)/*BL*(*t*). The intensity loss due to photobleaching, *BL*(*t*), was heuristically fitted with a (normalized) biexponential decay, *BL*(*t*) = *A* exp(-*t*/*τ*_1_) + (1 - *A*)exp(-*t*/*τ*_2_), with fractional amplitudes *A* and 1 – *A* and lifetimes *τ*_1_ and *τ*_2_.

##### Relocation of caveolar proteins after lesioning

As caveolar proteins may relocate into the cytosol after lesioning, we select (i) a region between two Z-lines close to the lesion (centered 2 – 5 μm away from the lesion spot) to quantify the cytoplasmic signal in zebrafish embryos, *I*_raw_(*t*), (ii) a region outside of the embryo to correct for background, *BG*, and (iii) a region inside the sample but far from the lesion site to correct for photobleaching, *BL* (all 20 × 20 pixels, ∼0.86 × 0.86 μm^2^). The final intensity, *I*(*t*), was determined as described above.

##### Single-molecule localization and tracking

The SMLM data were analyzed with the a-live PALM software^98^ to extract photon emission events from single fluorophores in each image frame, yielding locations and their uncertainty (standard deviation), from which super-resolved images were reconstructed. To reject erroneous events, the localization data were filtered in two ways. (1) Single-molecule spots with standard deviations of less than one pixel (109 nm) were rejected as well as spots with a localization precision >70 nm. To assemble trajectories of individual fluorophores, we used the ‘Track’ algorithm^99^ to link locations in successive camera frames, with the localization search radius (i.e., the maximum displacement between successive frames) set to 500 nm (Figure S6). The minimal length of a trajectory was set to five frames, and we did not allow for any gap (missing event) in a trajectory sequence.

For each trajectory, the mean square displacements (MSDs) were calculated according to ref. 100 as,

(Equation 1) $$MSD\left(t\right)=\left\langle {\left(x_{i+n}-x_{i}\right)}^{2}\right\rangle +\left\langle {\left(y_{i+n}-y_{i}\right)}^{2}\right\rangle =4Dt+4\sigma_{\text{loc}}^{2}\text{,}$$

where $x_{i}$ and $y_{i}$ are Cartesian coordinates of the molecule at the *i*^th^ position along the trajectory, and the angular brackets denote averages taken over all pairs of locations with the same time lag, *t* = *n* Δ*t*, with *n* being the difference in the position index (or camera frame number) and Δ*t* denoting the camera dwell time (30 ms). For simple Brownian diffusion, the MSD increases linearly with time, with the slope given by four times the diffusion coefficient, *D*. Importantly, the displacement from one frame to the next, i.e., within 30 ms, is on the nanometer scale for diffusion in membranes (Figure S6). Therefore, even though mEosFP*thermo*-based SMLM provides an excellent localization precision, σ_loc_ ≈ 30 nm, this uncertainty is still significant and must be included in the MSD calculation according to Equation 1.

To minimize the uncertainty in the determination of *D*, we took only those trajectories for the MSD analysis that extended over at least nine image frames and calculated MSDs only for time lags, *t*, with *n* ≥ 4 and at least four MSD values (Equation 1) for averaging. From the time dependence of the MSDs, linear regression with offset yielded the slope and thus the diffusion coefficient, *D*, of a particular fluorophore (Equation 1). In view of the localization uncertainties, it is not surprising that a fraction of the curves corresponding to very slowly moving or immobile molecules even displayed negative slopes. To include these events in our analysis, we simply took the modulus of *D* (Figure S7).

With ca. 8 frames on average (Figure S6), our single-molecule trajectories are very short due to the unavoidable photobleaching of the fluorescent protein marker. Consequently, the uncertainty in the MSD determination is large. It is well known that, for a system with a well-defined D, the *D* values extracted from short trajectories are distributed according to a Gaussian on a logarithmic scale, i.e., a log-Gaussian.^101^ Therefore, we define $\tilde{D}=\log\;[D/\left(1\;\mu m^{2}s^{-1}\right)$] and use the tilde to indicate parameters on a logarithmic scale.

Accordingly, we compiled the $\tilde{D}$ values from all trajectories of a given sample in a histogram with logarithmic bin widths (Δ $\tilde{D}$ = 0.2). These histograms were area-normalized to yield probability density functions (PDFs), quantifying the probability of measuring a particular $\tilde{D}$ value. Even though each individual measurement of *D* is imprecise, we can faithfully resolve different classes of moving entities from the histograms by fitting sums of log-Gaussian functions, as was shown previously,^102^ using,

(Equation 2) $$PDF\left(\tilde{D}\right)=\sum_{j}^{J}\frac{f_{j}}{{\tilde{\sigma }}_{j}\sqrt{2\pi }}\exp\left[\frac{\left(\tilde{D}-{\tilde{D}}_{j}\right)}{2{\tilde{\sigma }}_{j}^{2}}\right]\text{.}$$

Here, $f_{j}$, ${\tilde{D}}_{j}$, and ${\tilde{\sigma }}_{j}$ are the fractional areas (populations), center positions and standard deviations of *J* log-Gaussians, respectively. Thus, the PDF is generally specified by 3*J* – 1 free parameters (the “– 1” accounts for the normalization). To strengthen the significance of our analysis, we rigorously minimized the number of free parameters in the fit of multiple log-Gaussians by fitting several histograms in a single run and sharing the ${\tilde{D}}_{j}$, and $\sigma_{j}$ parameters between them if it appears physically sensible. This “Occam’s razor” approach aims to incorporate prior knowledge to reduce ambiguities of the fits. For example, one may reasonably expect that histograms before and after lesioning contain the same classes of moving entities, represented by log-Gaussians, but with different fractional weights. Indeed, the PDFs of Caveolin3 and Cavin1a (before and after membrane damage) could be fitted very well with three log-Gaussians, with their positions and variances taken as shared parameters in global fits of all four data sets. The variances of the three log-Gaussians were treated as a single parameter, as they mainly reflect the large relative uncertainties associated with MSD analysis of short trajectories.^101^ For TinyDysf, a fourth log-Gaussian was included to represent its fast free diffusion in the membrane. For LactC2, GPI and CAAX, a fifth log-Gaussian was necessary, which allowed us to model the different dynamics of the freely diffusing populations. The peak value, *D*_pk_, of the fast-diffusing population (Table S3) was determined by fitting a Gaussian function to histogram points with amplitudes >50% of the maximum.

For each sample, we analyzed up to ca. 70,000 trajectories extracted from measurements on 3 – 6 different days, with 5 – 15 cells on each day (Table S3). In the table, we have also compiled the best-fit parameters from fitting multiple log-Gaussians including their uncertainties.

##### Quantification of membrane and Z-line fluorescence

The relative fluorescence in the Z-line of individual muscle cells in wildtype or Δcavin1a embryos was measured, taking the intensity in the sarcolemma as an internal reference. For each cell, the mean intensities of the sarcolemma and the Z-line were taken at 10 different positions in each muscle cell using ImageJ. A rectangular ROI was drawn, excluding the T-tubule attachment points within the sarcolemma (placing the ROI onto the sarcolemma between two Z-line/T-tubule attachment points), and the mean intensity values of both were copied to Microsoft Excel 2007 for statistical analysis. For each cell, the mean of the 10 mean intensities of the sarcolemma and the Z-line were calculated. Finally, the mean intensity of the Z-line was divided by the mean intensity of the sarcolemma, thereby comparing the fluorescence signal in the Z-line (indirectly representing the amount of fluorescent protein in this compartment) between wildtype and knock-out samples (n ≥ 6).

### Quantification and statistical analysis

Details of the statistical tests that were used and the statistical parameters are reported in the figure legends. Statistical parameters were calculated with Origin Pro 2022 (Origin lab).
