# Supplementary material for: Caveolae disassemble upon membrane lesioning and foster cell survival
Source: iScience. 2024 Jan 9;27(2):108849. doi: 10.1016/j.isci.2024.108849 (PMC10831942; doi:10.1016/j.isci.2024.108849)
Supplement: Document S1. Figures S1–S7 and Tables S1–S3 [file mmc1.pdf]

iScience, Volume *27*

Supplemental information

Caveolae disassemble upon membrane lesioning and foster cell survival

Martin Štefl, Masanari Takamiya, Volker Middel, Miyase Tekpınar, Karin Nienhaus, Tanja Beil, Sepand Rastegar, Uwe Strähle, and Gerd Ulrich Nienhaus

SUPPLEMENTAL FIGURES

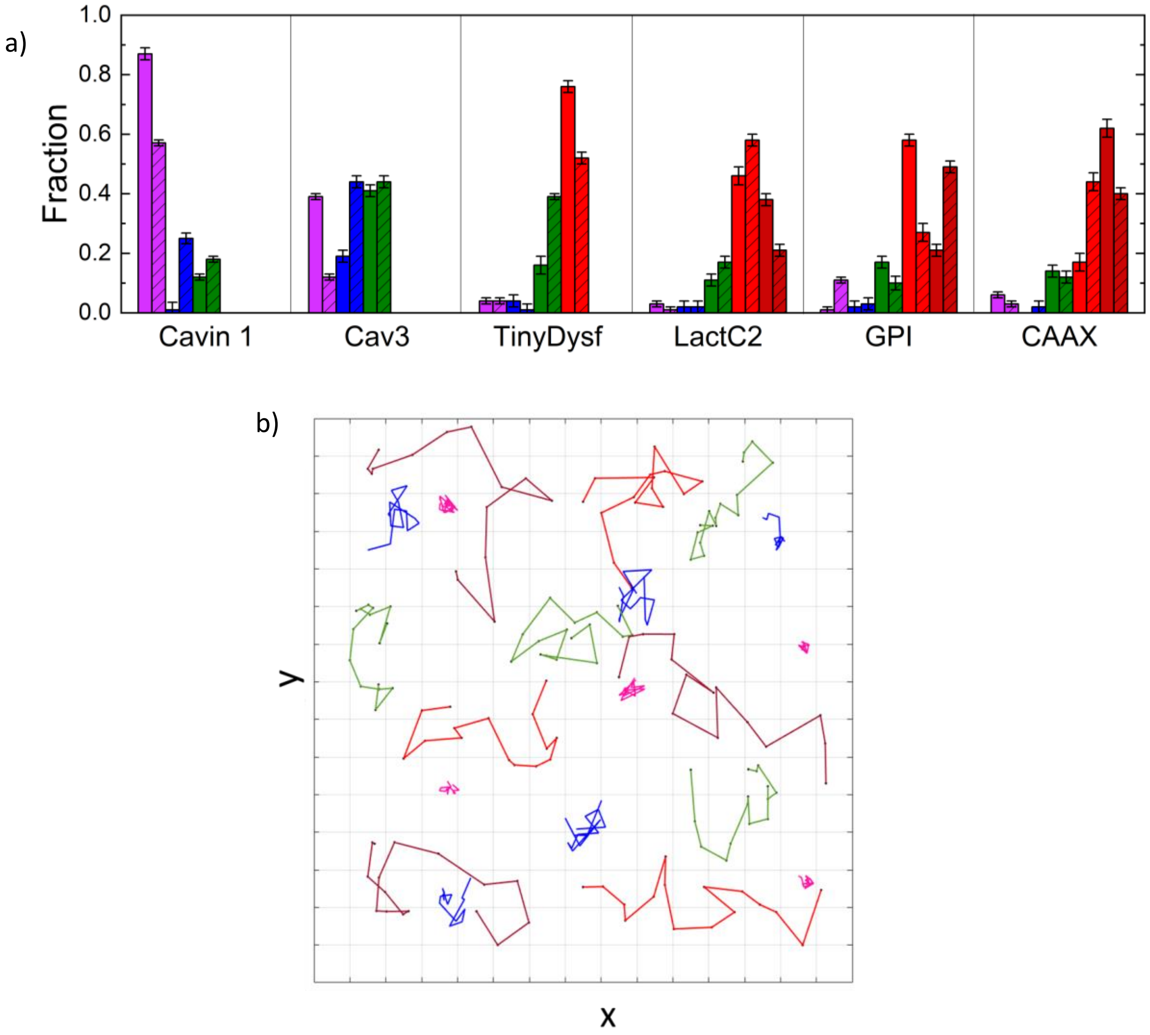

Figure S1. Diffusional dynamics of mEosFP*thermo* fusion proteins, related to Figures 4 and 5. (a) Graphical depictions of the fractional weights of the different diffusing populations obtained by fitting multiple log-Gaussians to the PDFs in Figures 4 and 5 (numerical data in Table S3). The log-Gaussians are centered on (average diffusivity) ~0.002 $\mu m^2 s^{-1}$ (magenta), ~0.014 $\mu m^2 s^{-1}$ (blue), ~0.07 $\mu m^2 s^{-1}$ (green), ~0.27 $\mu m^2 s^{-1}$ (red), and ~0.60 $\mu m^2 s^{-1}$ (dark red) determined before (plain bars) and after (hatched bars) lesioning. Error bars indicate uncertainties provided by the fitting routine. **(b)** Exemplary trajectories calculated from TinyDysf-mEosFP*thermo* localizations in successive SMLM image frames (15 – 17 frames each). Color code as in (a) to indicate the different mobility classes. Grid spacing, 200 nm.

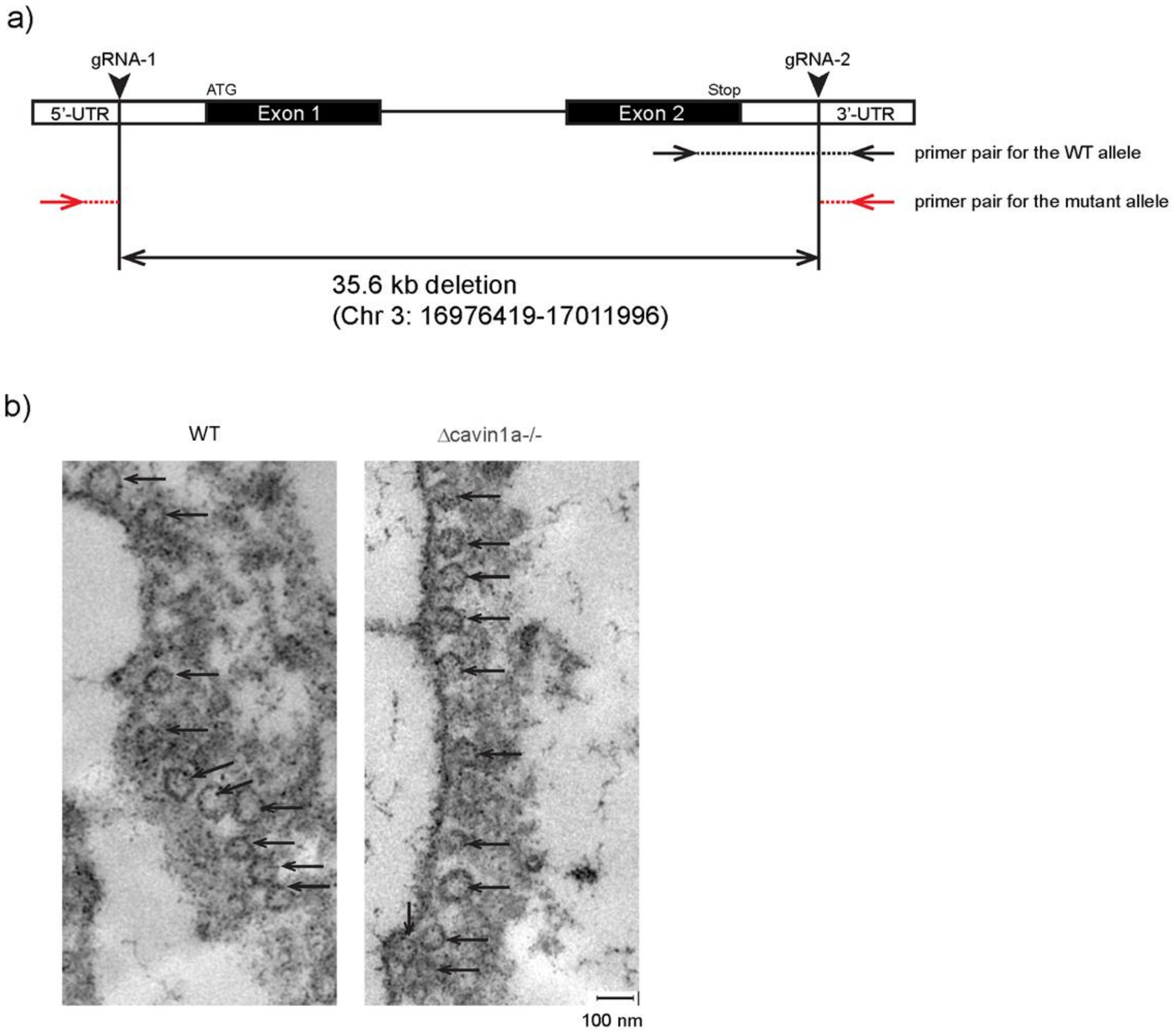

Figure S2. **Characterization of the Δcavin1a zebrafish mutant, related to Figure 6. (a)** Schematic illustration of the knock-out strategy. The CRISPR/Cas9 method with two different guide RNAs (arrowheads; gRNA-1 [5'-AGACTTGAGGGAAAGAGGAAGG] and gRNA-2 [5'-ATAATTCACACCCTGGGGTACA]) was used to delete the entire protein coding region of the cavin1a protein (35.6 kb deletion; Chr 3: 16976419-17011996). Zygosity of *cavin1a* mutants was confirmed by genomic PCR using two sets of primers, one for detecting the presence of the unmodified WT allele (black arrows for primers; dotted black line for the PCR product) and another for the mutant allele with deletion (red arrows for primers; dotted red line for the PCR product). Homozygous mutation was confirmed by the absence and the presence of PCR products from the WT and mutant alleles, respectively. **(b)** TEM images of caveolae in the notochord of WT and Δcavin1a zebrafish, acquired with ruthenium red-stained specimens (20,000× magnification).

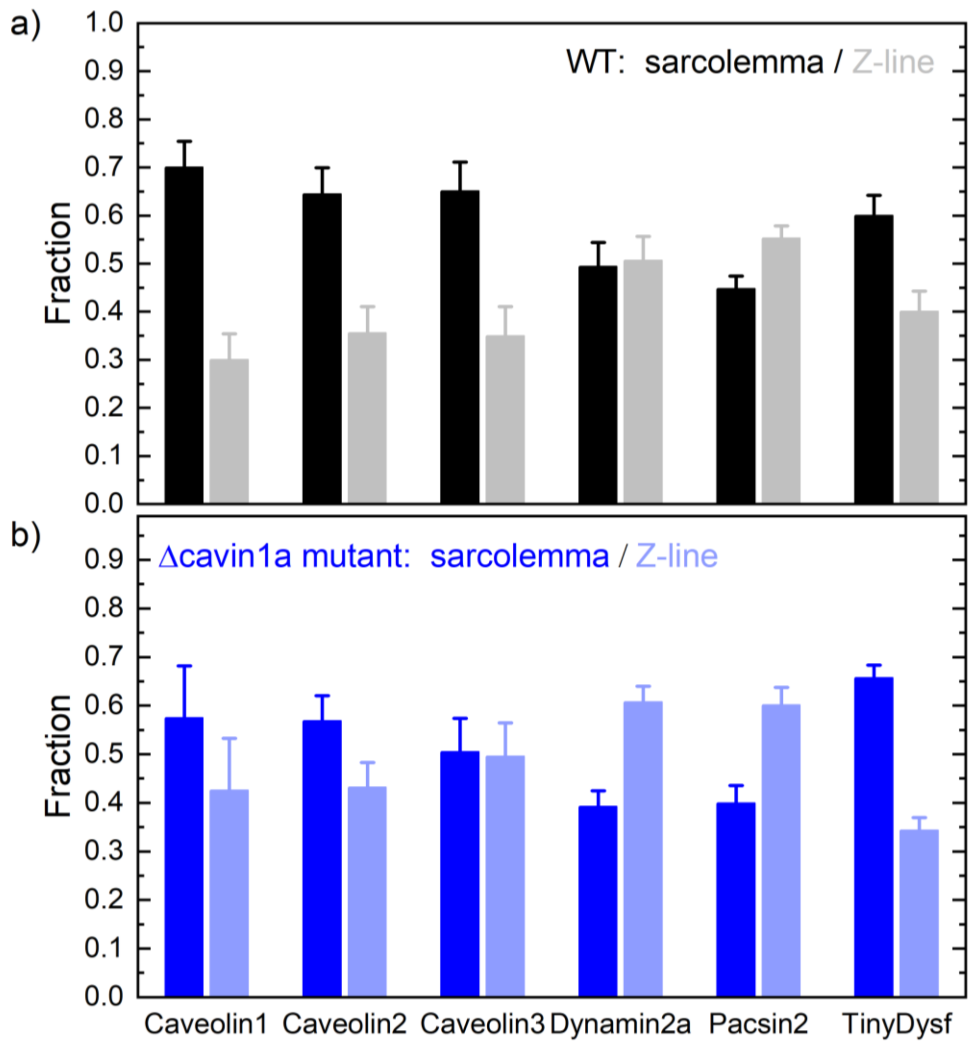

Figure S3. Fractions of Caveolin1, -2, -3, Dynamin2a, Pacsin2 and TinyDysf in the sarcolemma and the Z-lines of zebrafish cells, related to Figure 6. Fractions were calculated from the emission intensities I_{PM} and $I_{Z\text{-line}}$ as $I_{PM}/(I_{PM} + I_{Z\text{-line}})$ and $I_{Z\text{-line}}/(I_{PM} + I_{Z\text{-line}})$, respectively**. (a)** WT cells. **(b)** Δcavin1a zebrafish mutant cells. Bars represent average fractions from multiple experiments (6 – 21 cells), errors indicate the SD.

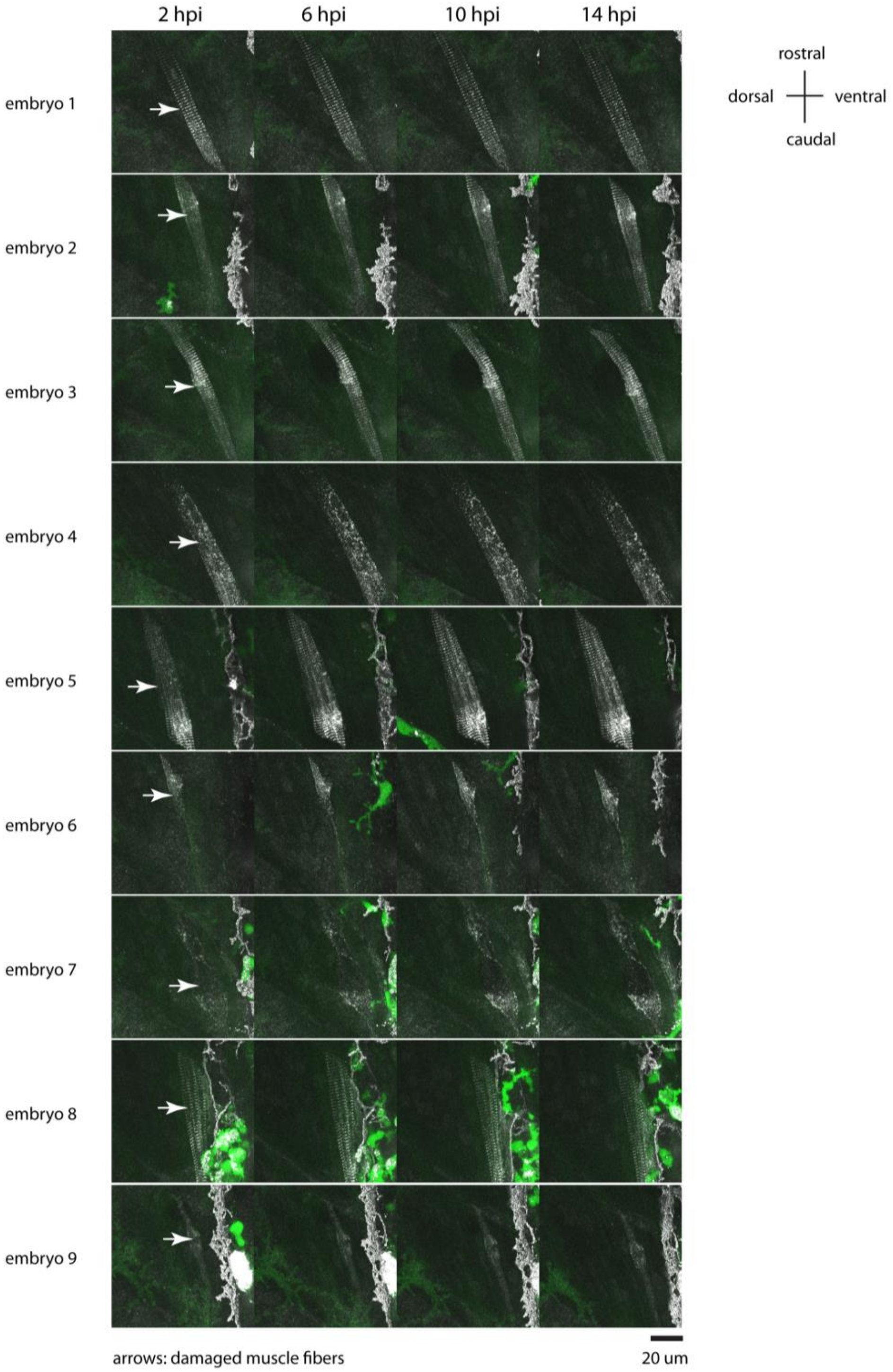

Figure S4. Probing long-term survival of zebrafish embryos (genotype *cavin1a+/+, Tg(mpeg1.1:gfp*) after laser-induced lesioning, related to Figure 7. Maximum intensity projection views of individual fibers (embryos) at 2, 6, 10, and 14 hours post injury (hpi). Shown are overlays of the confocal reflection of 488-nm laser light (gray) and GFP emission originating from macrophages. Damage sites are marked by arrows. All cells survived.

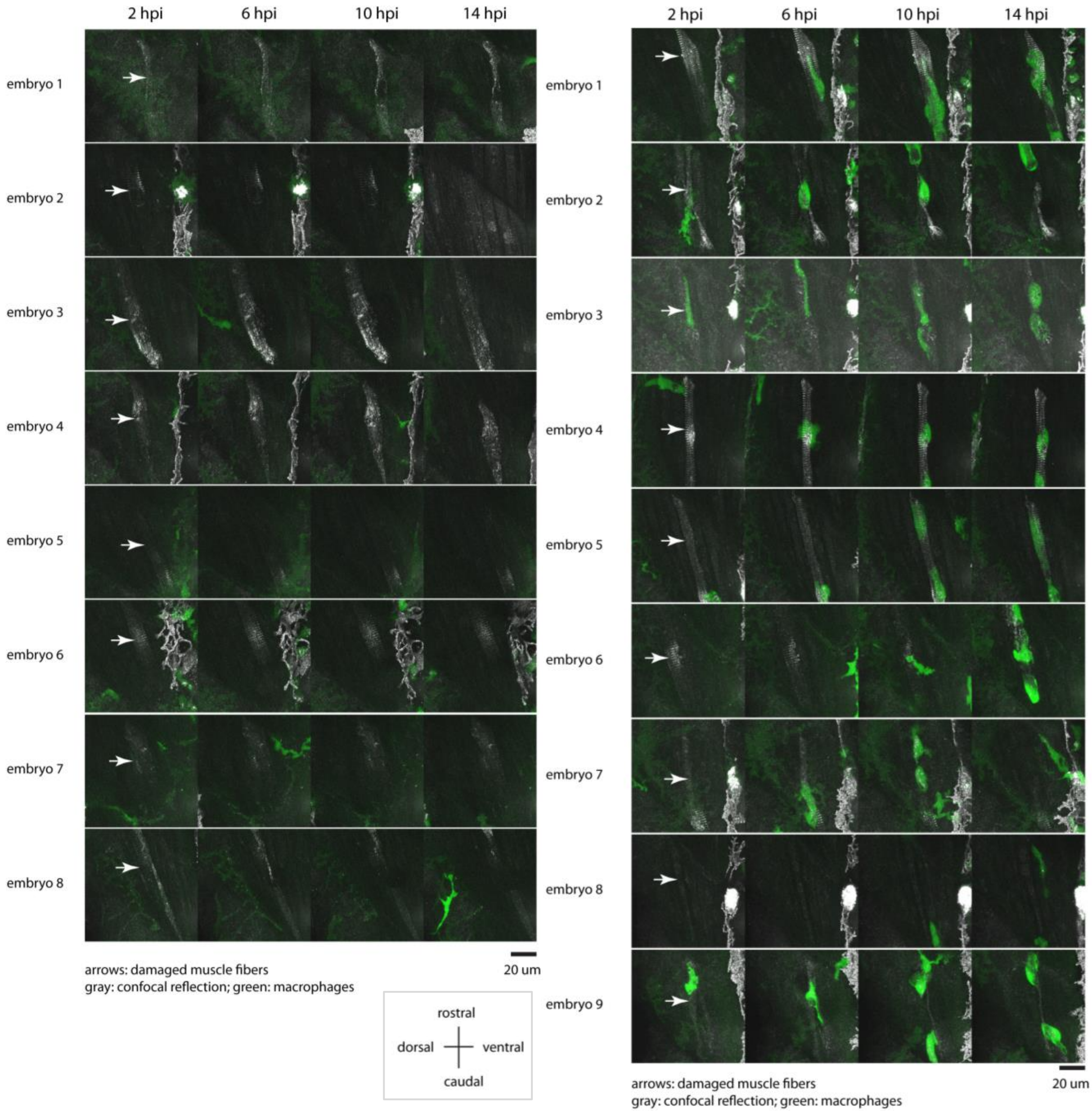

Figure S5. Probing long-term survival of zebrafish embryos (genotype *cavin1a-/-, Tg(mpeg1.1:gfp*) after laser-induced lesioning, related to Figure 7. Maximum intensity projection views of individual fibers (embryos) at 2, 6, 10, and 14 hpi. Shown are overlays of the confocal reflection of 488-nm laser light (gray) and GFP emission originating from macrophages. Damage sites are marked by arrows. Left: surviving cells. Right: dead cells that were eaten up by macrophages. Nine out of 17 cells (53%) did not survive.

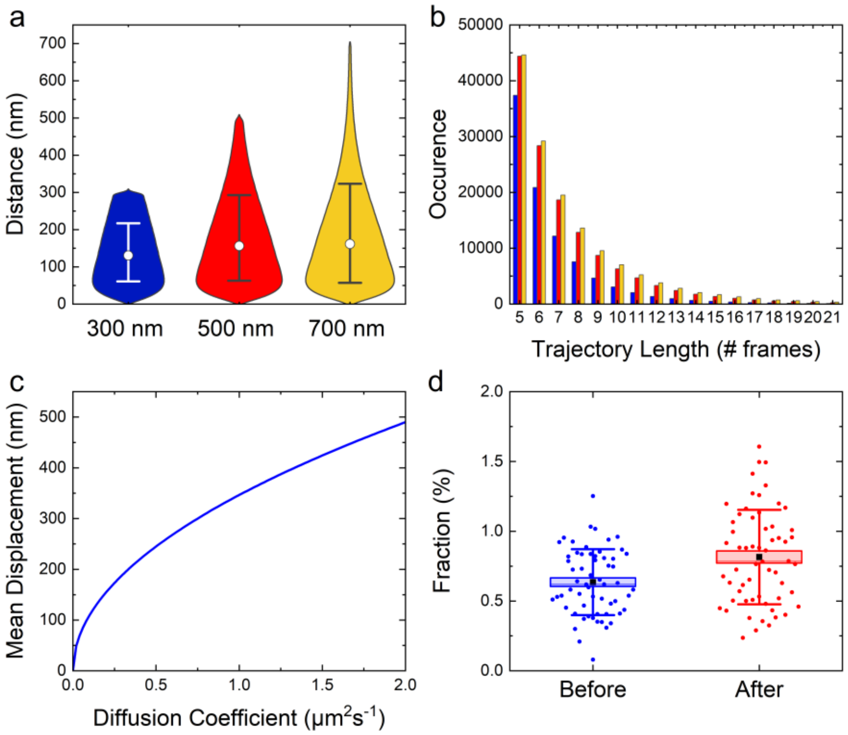

Figure S6. Determination of the search radius for the assembly of trajectories, related to Figures 4 and 5. (a) Distribution of distances between CAAX localizations observed in successive frames, with the search radius (maximum displacement) set to 300, 500 and 700 nm. With respect to 700 nm search radius, the number of frames per trajectory was reduced by 19% and 3% for values of 300 and 500 nm, respectively. **(b)** Histogram of trajectory lengths, determined from measurement of six cells expressing TinyDysf, with the search radius between successive frames set to ≤300 nm (blue), ≤500 (red) nm and ≤700 nm. Average lengths are 6.8, 7.5, 7.7 frames for the three search radii, respectively. **(c)** Root-mean-square displacement ($\sqrt{4Dt}$) of freely diffusing particles, calculated as a function of the diffusion coefficient, D, using a time lag t = 30 ms that corresponds to our image dwell time. **(d)** Fraction of CAAX localizations with another localization within a distance of 500 nm, plotted before and after damage for measurements on 60 cells (data points). The small fractions (average below 1%) ensure that the likelihood of combining trajectories from two CAAX constructs is small.

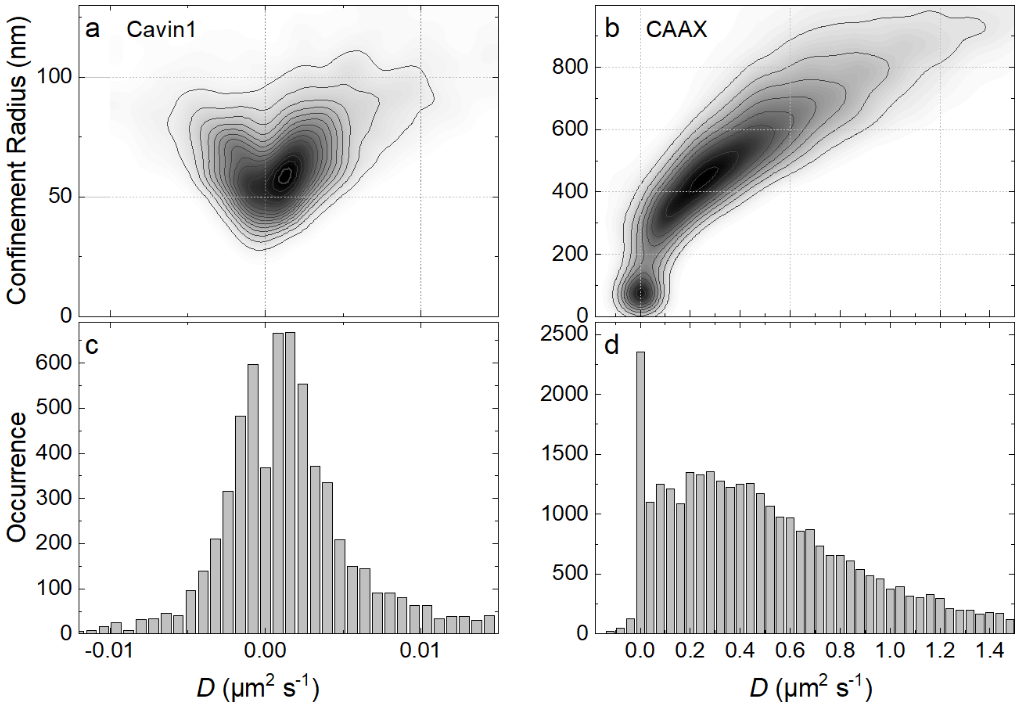

Figure S7. MSD analysis of the trajectories of essentially immobile molecules may yield negative diffusion coefficients, related to Figures 4 and 5. Two-dimensional contour plot of the number of trajectories, plotted against the confinement radius[1,2] and the diffusion coefficient, *D*, for **(a)** Cavin1 and **(b)** CAAX. The confinement radius corresponds to the maximum variance value in either the x or y positions of the trajectories, max(σ_{xx}, σ_{yy}). A Gaussian kernel was used for smoothing. Bandwidths were determined by using Silverman's rule.[2] **(c, d)** Histograms of occurrences with particular diffusion coefficients corresponding to (a, b), respectively.

[1] Vega, A.R., Freeman, S.A., Grinstein, S., and Jaqaman, K. (2018). Multistep Track Segmentation and Motion Classification for Transient Mobility Analysis. Biophys. J. 114, 1018-1025.

[2] Silverman, B.W. Density Estimation for Statistics and Data Analysis (Springer New York, NY, 1986).

SUPPLEMENTAL TABLES

Table S1. Primers used for cloning (related to KRT).

Primer	Sequence (5' to 3' direction)	Comments
**Fusion vectors**		
F2.mGarnet2-C.SpeI	ATACTAGTGCGGCCGCTCGAGATGGTTCAGCAGGTTCTGCAGCAGGTTCTGGAGAGTTCCTCGAGAACAGCCTGATCAAAGAAAACATG	C-terminal fusion of mGarnet2 in pME backbone
R2.mGarnet2-C.SacI	ATGAGCTCGGCTTACCCTCCGCCCAGG	
F.LinkNxhoI	GCGCTCGAGGCCGGTGGCGGAGGGTCTGGAG	linker for N-terminal fusion into the pME backbone
R.LinkNclaI	TTATCGATGGCTGATCCACCCCCGCCG	
F.CloC.Link.SpeI	CGAACTAGTGGCGGTGGCGGAGGGTCTGGAGGGGGCGGTTCCGGCGGGGGTGGATCAGTGAGCAAGGGCGAGGAGCTGTTCAC	C-terminal fusion of Clover in the pME backbone
R.CloC.SacI	GCGGAGCTCGCCCTAGCATTTAGGTGACACTATAGAATAG	
F.EosFPNkpnI	GCCAGGTACCCCGCCACCATGAGTGCGATTAAGCCAGACATG	N-terminal fusion of mEosFP*thermo* in the pME backbone
R.EosFPNxhoI	GCCACTCGAGGGCCCGTCTGGCATTGTCAGG	
F. GcAMP5A	CAAATGATGACAGCGAAGAAGCTGAACCCTCCTGATGAGAGTGGCCCCGGCTGCATGAGCTGCAAGTGTGTGCTCTCCTAAGGCGGATCCACTAGT	N-terminal fusion of CAAX to the calcium sensor
R. GcAMP5A	ACTAGTGGATCCGCCTTAGGAGAGCACACACTTGCAGCTCATGCAGCCGGGGCCACTCTCATCAGGAGGGTTCAGCTTCTTCGCTGTCATCATTTG	

Cloning of cDNAs

F.LactC2GFP.ClaI	GCATCGATGCGGCCACCATGGTGAGCAAGGGCGAG	LactC2:GFP into pME backbone
R.LactC2GFP.XhoI	CGACTCGAGGGCCTAACAGCCCAGCAGCTCCAC	
F.LactC2N.ClaI	GCATCGATGCCTGCACTGAACCCCTAGGCCTG	N-terminal LactC2 fusion vectors into pME backbone
R.LactC2N.SpeI	GCACTAGTGGCCTAACAGCCCAGCAGCTCCAC	
F.TM-C	GCGATCGATGCCACCATGTGGCTTATTCTGGGCCTCTTTATAC	zfTM-C into pME backbone
R.TM-C	GCCACTAGTGGCCTGTGTTCCCTTTCCTAG	
F.Cav1.KpnI	ATGGTACCGCCGCCACCATGACTAGCGGATACAAGGAC	Caveolin 1-C into pME backbone
R.Cav1.SpeI	ATACTAGTGGCCACCACCTTAGTAGCAGTGAC	
F.Cav2.KpnI	ATGGTACCGCCGCCACCATGGGTCTAGAAAAAGAAAAATC	Caveolin 2-C into pME backbone
R.Cav2.SpeI	ATACTAGTGGCGATTTGCTCAGTCTTGATGTTG	
F.Cav3.KpnI	ATGGTACCGCCGCCACCATGGCGGACCAGTACAACAC	Caveolin3-C into pME backbone
R.Cav3.SpeI	GGACTAGTCGCAACCTCTTTGCGTAAGGC	
F.EHD2a.C.KpnI	ATACTAGTGGCATCATCGTCGGTAATCTCAGAG	EHD2a-C into pME backbone
R.EHD2a.C.SpeI	AT GGTACC GCCACC ATGTCTGCCAAAGGAATGAGG	
F2.Cavin4a.KpnI	GGGGTACCGCCACCATGGAAAAGAGAGGAGATGTCATATT	Cavin4a-C into pME backbone
R2.Cavin4a.SpeI	GGACTAGTCGCGTCCTCATGGATACGAGTTGC	
F.Cavin4b.KpnI	ATGGTACCGCCACCATGGCTGATAAACTGGGACTG	Cavin4b-C into pME backbone
R.Cavin4b.SpeI	ATACTAGTGGCAGAAAGCTGCTTCATATCATACATC	
F.Pacsin2.KpnI	ATGGTACCGCCGCCACCATGTCGGATTTCAACGACTCTC	Pacsin2-C into pME backbone
R2.Pacsin2.BamHI	ATGGATCCGGCCTGCAGATCCTCCACATAGTTG	

F2.Dnm2a.KpnI	ATGGTACCGCCGCCACCATGGGCAACCGGGGGATG	Dnm2a-C into pME backbone
R.Dnm2a.SpeI	ATACTAGTGGCGTCTAGCAGGGAGGGCTCAG	
F.unc45b195BamHI	CAGAGGATCCGCGCTTAATGGTTTCTTACAGTA	subclone unc45b.195 promoter into the p5E backbone
R.unc45b195SacII	GCTGCCGCGGGCGATAGGGTCTATTTATGGAG	
F.CAAXEosFPNClaI	GCCAATCGATGCCAAGCTGAACCCTCCTGATGA	to fuse CAAX N-terminally to mEosFP*thermo* into the pME backbone
R.CAAXEosFPNSpeI	GCGACTAGTGGCTCAGGAGAGCACACACTTGC	
F.Ca-Report.HindIII	GGCCAAGCTTGCCACCATGGGTTCTCATCATCATC	subclone GCaMP5A into pME backbone
R.Ca-Report.BamHI	GCGGGATCCGCCTTACTTCGCTGTCATCATTTG	

Mutagenesis

F.Clo.KpnI-mut	AGGACGACGGTACGTACAAGACCCGCG	to mutate a KpnI site within Clover (silent mutation)
R.Clo.KpnI-mut	CGCGGGTCTTGTACGTACCGTCGTCCT	
F.unc195.del	CCTCTCCATAAATAGACCCTATCACAAGTTTGTACAAAAAAGTTGAAC	to delete a short sequence 3' of the unc45b.195 within the p5E
R.unc195.del	GTTCAACTTTTTTGTACAAACTTGTGATAGGGTCTATTTATGGAGAGG	
F.WRRFK	TATCGATGCCACCATGTGGAGGAGGTTTAAGTGGCTTATTCTGGGCC	to insert the WRRFK sequence into the TM-C backbone
R.WRRFK	GGCCCAGAATAAGCCACTTAAACCTCCTCCACATGGTGGCATCGATA	

CRISPR/Cas9

guideRNA1_Exon1 (*cavin1a*)	GGCCCAGTGTGCAAGAACGC	sequence of the targeting gRNA without PAM
guideRNA2_Exon2 (*cavin1a*)	GGAAGGTGTAAAGTCAGTGG	
T7guideRNA-oligo_Exon1 (*cavin1a*)	TAATACGACTCACTATAGGCCCAGTGTGCA AGAACGCGTTTTAGAGCTAGAAATAGCAAG	T7-sequence of the targeting gRNA without PAM-overlapping sequence to constant oligo
T7guideRNA-oligo_Exon2 (*cavin1a*)	TAATACGACTCACTATAGGAAGGTGTAAAG TCAGTGGGTTTTAGAGCTAGAAATAGCAAG	
Constant oligo	AAAAGCACCGACTCGGTGCCACTTTTTCAA GTTGATAACG GACTAGCCTTATTTTAACTTGCTATTTCTAG CTCTAAAAC	annealed with T7guideRNA-oligo to make cloneless gRNA
Mutant allele	left: agacttgagggaaagaggaagg<br>right: ataattcacaccctggggtaca	Primer used for genotyping (cavin1a mutants)
WT allele	left: ggaagaagctgaactggtcag<br>right: ataattcacaccctggggtaca	

Table S2 Summary of Gateway entry clones for constructing the expression vectors (related to KRT)

**Gateway recombined expression vector**	**p5E entry clone (with internal reference number)**	**pME entry clone (with internal reference number, if available)**
unc45b:Clover-cavin1a (#277)	*p5E-unc45b.195bp*[a] *(#169)*	*pME- Clover-cavin1a*
unc45b:cavin4a-Clover (#216)	*p5E-unc45b.195bp (#169)*	*pME- cavin4a-Clover (#112)*
unc45b:cavin4b-Clover	*p5E-unc45b.195bp (#169)*	*pME- cavin4b-Clover*
unc45b:EHD2a-Clover	*p5E-unc45b.195bp (#169)*	*pME- EHD2a-Clover (#111, #138*
unc45b:caveolin1-Clover	*p5E-unc45b.195bp (#169)*	*pME- caveolin1-Clover (#98)*
unc45b:Clover-caveolin3	*p5E-unc45b.195bp (#169)*	*pME- caveolin3-Clover (#100)*
unc45b:pacsin2-Clover (#253/298)	*p5E-unc45b.195bp (#169)*	*pME- pacsin2-Clover (#126)*
unc45b:tinyDysf-Clover (#345)	*p5E-unc45b.195bp (#169)*	*pME- tinyDysf-Clover*
unc45b:caveolin2-Clover	*p5E-unc45b.195bp (#169)*	*pME- caveolin2-Clover (#99)*
unc45b:dynamin2a-Clover (#390)	*p5E-unc45b.195bp (#169)*	*pME- dynamin2a-Clover (#114)*
unc45b:EGFP-LactC2 (#250)	*p5E-unc45b.195bp (#169)*	*pME- EGFP-LactC2 (#51)*
unc45b:Annexin2a-mOrange	*p5E-unc45b.195bp (#169)*	*pME- Annexin2a-mOrange*
unc45b:Annexin6-mOrange	*p5E-unc45b.195bp (#169)*	*pME- Annexin6-mOrange*[b]
unc45b:mEosFPthermo-LactC2	*p5E-unc45b.195bp (#169)*	*pME- mEosFPthermo-LactC2 (#61)*
unc45b:tinyDysf-mEosFPthermo	*p5E-unc45b.195bp (#169)*	*pME- tinyDysf-mEosFPthermo (#64)*
cmv:caveolin3-mGarnet2	*p5E-cmv/sp6 (Tol2ki t#382)*	*pME- caveolin3-mGarnet2 (#139)*
cmv:mGarnet2-cavin1a (#276)	*p5E-cmv/sp6 (Tol2kit #382)*	*pME- mGarnet2-cavin1a (#110)*
cmv:tinyDysf-Clover	*p5E-cmv/sp6 (Tol2kit #382)*	*pME- tinyDysf-Clover*
cmv:caveolin3-mEosFPthermo	*p5E-cmv/sp6 (Tol2kit #382)*	*pME- caveolin3-mEosFPthermo (#128)*
cmv:CAAX-mEosFPthermo (#418)	*p5E-cmv/sp6 (Tol2kit #382)*	*pME- CAAX-mEosFPthermo (#59)*
cmv:tinyDysf-mEosFPthermo	*p5E-cmv/sp6 (Tol2kit #382)*	*pME- tinyDysf:mEosFPthermo (#64)*
cmv:mEosFPthermo-LactC2 (#411)	*p5E-cmv/sp6 (Tol2kit #382)*	*pME- mEosFPthermo-LactC2 (#61)*

[a] unc45b 195 bp minimal promoter sequence (with primers underlined):
GCTTAATGGTTTGTTACAGTATGTGTGTAACACCTTCCCCCCCAGCCCAGCTCTCCACTTTTCTCATTCTAGAA
AGTACCAGTCAACTCTCCACCAGCCCAGCTGTTGGCAGACGCACACCTCCATCCCCCTGCCCTCAGACATTTG
CCACTGATTTCTCAGCTGTCATCCCCTCTCCATAAATAGACCCTATCA

[b] Roostalu, U., and Strähle, U. (2012). In Vivo Imaging of Molecular Interactions at Damaged Sarcolemma. Dev. Cell 22, 515-529 .

Table S3. **SMLM data collection statistics, parameters of histogram fits with multiple log-Gaussians, peak positions (related to Figures 4 and 5).**

	**Caveolin3**		**Cavin1**		**TinyDysf**		**LactC2**		**GPI**		**CAAX**			
**Overall statistics of data collection**														
**cells per day**	9 / 7 / 12 / 12 / 10 / 5		6 / 11 / 15		18 / 14 / 9 / 6		11 / 16 / 11		7 / 13 / 10		15 / 10 / 15 / 9 / 11			
**lesioning**	before	after	before	after	before	after	before	after	before	after	before	after		
**# trajectories**[a]	58,002	73,668	7,178	8,931	33,552	14,136	57,698	64,956	3,272	3,620	30,980	33,609		
**Parameters of histogram fits with multiple log-Gaussians**														
j	**fractions, f_j**												**log [D_j / (µm^2 s^{-1})]**	**log [σ_j / (µm^2 s^{-1})]**
1	0.39 ± 0.01	0.12 ± 0.01	0.87 ± 0.02	0.57 ± 0.01	0.04 ± 0.01	0.08 ± 0.01	0.03 ± 0.01	0.01 ± 0.01	0.11 ± 0.01	0.10 ± 0.01	0.06 ± 0.01	0.03 ± 0.01	-2.626 ± 0.009	0.431 ± 0.008
2	0.19 ± 0.02	0.44 ± 0.02	0.01 ± 0.02	0.25 ± 0.02	0.04 ± 0.02	0.01 ± 0.02	0.02 ± 0.02	0.02 ± 0.02	0.03 ± 0.02	0.01 ± 0.02	0.00 ± 0.02	0.02 ± 0.02	-1.861 ± 0.027	0.431 ± 0.008
3	0.41 ± 0.02	0.44 ± 0.02	0.12 ± 0.01	0.18 ± 0.01	0.16 ± 0.03	0.39 ± 0.01	0.11 ± 0.02	0.17 ± 0.02	0.10 ± 0.02	0.16 ± 0.02	0.14 ± 0.02	0.12 ± 0.02	-1.141 ± 0.020	0.431 ± 0.008
4	–	–	–	–	0.76 ± 0.02	0.52 ± 0.02	0.46 ± 0.03	0.58 ± 0.02	0.27 ± 0.03	0.22 ± 0.03	0.17 ± 0.03	0.44 ± 0.03	-0.568 ± 0.007	0.320 ± 0.006
5	–	–	–	–	–	–	0.38 ± 0.02	0.21 ± 0.02	0.49 ± 0.02	0.49 ± 0.02	0.62 ± 0.03	0.40 ± 0.02	-0.224 ± 0.008	0.261 ± 0.006
**Peak position of the freely diffusing component**														
**log [$\widetilde{D}_{pk}$ / (µm^2 s^{-1})]**					-0.57 ± 0.02	-0.71 ± 0.03	-0.37 ± 0.03	-0.48 ± 0.02	-0.32 ± 0.022	-0.29 ± 0.02	-0.26 ± 0.02	-0.39 ± 0.02		
**$\widetilde{D}_{pk}$ (µm^2 s^{-1})**					0.27	0.19	0.43	0.33	0.48	0.51	0.55	0.41		

[a] with nine or more camera frames.
